# Antibiotic dose-response curves can measure antibiotic activity against *Mycobacterium abscessus* and *Mycobacterium peregrinum*

**DOI:** 10.1128/aac.01876-25

**Published:** 2026-04-06

**Authors:** Husain Poonawala, Kathleen Davis, Myles E. Kenny, Ares Alivisatos, Nhi Van, Tracy Washington, Vinicius Calado Nogueira de Moura, Charles L. Daley, Bree B. Aldridge

**Affiliations:** 1 Division of Geographic Medicine and Infectious Diseases, Department of Medicine, Tufts Medical Center 1867, Boston, Massachusetts, USA; 2 Department of Pathology and Laboratory Medicine, Tufts Medical Center 1867, Boston, Massachusetts, USA; 3 Stuart B. Levy Center for Integrated Management of Antimicrobial Resistance, Boston, Massachusetts, USA; 4 Department of Molecular Biology and Microbiology, Tufts University School of Medicine 12261, Boston, Massachusetts, USA; 5 Divison of Mycobacterial and Respiratory Infections, Department of Medicine, National Jewish Health 551774 , Denver, Colorado, USA; 6 Department of Medicine, University of Colorado School of Medicine 12225, Aurora, Colorado, USA; 7 Department of Biomedical Engineering, Tufts University School of Engineering 98044, Medford, Massachusetts, USA; Queen Mary University of London, London, United Kingdom

**Keywords:** multidrug resistance, antimicrobial activity, clarithromycin, inducible resistance, antimicrobial susceptbility testing, dose-response curves, *Mycobacteroides abscessus*, *Mycobacterium abscessus*

## Abstract

*Mycobacterium abscessus* is a drug-resistant pathogen associated with poor clinical outcomes despite prolonged treatment with multidrug antibiotic regimens. Apart from clarithromycin, antimicrobial susceptibility testing (AST) results for *Mycobacterium abscessus* cannot guide antibiotic selection. AST involves measuring the minimum inhibitory concentration (MIC), which is allowed to span a fourfold range in concentration. This accepted variability of the MIC limits the clinical utility of AST. Antimicrobial dose-response curves, obtained by measuring the growth inhibition of a given organism to increasing concentrations of an antibiotic, can yield metrics of antibiotic activity that are less variable than the MIC. We used Clinical and Laboratory Standards Institute growth conditions for rapidly growing nontuberculous mycobacteria AST to generate 990 dose-response curves across three time points (72 h, 96 h, and 120 h) for six guideline-recommended (clarithromycin, amikacin, cefoxitin, linezolid, tigecycline, and clofazimine) and five new (omadacycline, tedizolid, SPR719, SQ109, and bedaquiline) antibiotics against *Mycobacterium abscessus* subspecies *abscessus* ATCC 19977 and *Mycobacterium peregrinum* ATCC 700686. We established the fit of the dose-response curve (*R*^2^) as a quality control metric. Using the geometric standard deviation and median coefficient of variation, we demonstrated that the IC_50_ and IC_75_ (antibiotic concentrations corresponding to 50% and 75% growth inhibition, respectively) are less variable than the MIC. We identified time-dependent changes in dose-response curve metrics that allow the detection of inducible clarithromycin resistance with only 5 days of incubation. This study demonstrates the potential of dose-response curves in measuring antibiotic activity against *Mycobacterium abscessus*.

## INTRODUCTION

*Mycobacterium abscessus* (MAB) is a highly drug-resistant nontuberculous mycobacteria (NTM) responsible for difficult-to-treat pulmonary and extrapulmonary infections (1, 2). The 2020 multi-society guidelines for the treatment of NTM pulmonary disease recommend treatment with a combination of two to six drugs, with the caveat that “the optimal drugs, regimens, and duration of therapy are not known” (1, 3). Most patients will experience side effects from one or more antibiotics, often resulting in treatment cessation; fewer than half will attain microbiological cure (4–11).

MAB has three subspecies—*Mycobacterium abscessus* subspecies *abscessus*, *Mycobacterium abscessus* subspecies *massiliense*, and *Mycobacterium abscessus* subspecies *bolletti*—with differing clarithromycin susceptibility patterns depending on the activity of the *erm*(41) gene (3). Clarithromycin resistance is associated with treatment failure (1, 12). MAB subspecies *bolletti* and *abscessus* sequevars I, VI, and VII (80%–85% of MAB subspecies *abscessus*) have inducible resistance mediated by a functional *erm*(41) gene (13). The remaining 15%–20% of MAB subspecies *abscessus* harbor a T28C mutation in *erm*(41), while MAB subspecies *massiliense* has a truncated *erm*(41); both mutations render the gene nonfunctional and result in clarithromycin susceptibility.

Treatment regimens for MAB are designed using the results of antimicrobial susceptibility testing (AST) performed by broth microdilution according to Clinical and Laboratory Standards Institute (CLSI) guidelines for rapidly growing NTMs (3, 14). However, with the exception of clarithromycin, MAB AST results are not predictive of clinical outcomes (8, 14). Demonstration of clarithromycin susceptibility is essential for treatment and involves subspecies identification (*rpoB* sequence), and ruling out both inducible resistance (*erm*(41) sequence or 14-day incubation) and acquired resistance (*rrl* sequence or 5-day incubation)—these methods are usually available only in reference laboratories (14, 15). Of the remaining six drugs recommended for the treatment of MAB, AST results are either not predictive of treatment outcomes (amikacin, cefoxitin, and linezolid), unreliable due to instability (imipenem), or without interpretive criteria (clofazimine and tigecycline) (1, 15–18). There are no breakpoints for omadacycline, tedizolid, or bedaquiline, newer drugs that are promising for the treatment of MAB infections (1, 19, 20). The complexity and inadequacy of current AST methods contribute to the lack of progress against MAB (1).

AST involves measuring the minimum inhibitory concentration (MIC)—the lowest antibiotic concentration required to limit visible growth of an organism—and is specific to any antibiotic-bacterial pair (21). MICs are measured with twofold increases in concentration, and because measuring them involves numerous sources of error, repeat measurements can be one 2-fold measurement above or below the original measurement (22). For isolates with MICs close to the breakpoint for a given antibiotic, a repeat measurement that is one doubling higher or lower could change the interpretation from susceptible to resistant and vice versa. This change in classification was seen in 3%–24% of resistant *Enterobacterales* clinical isolates that underwent repeat testing (23). A post hoc analysis of the MERINO trial that took into account variability of the piperacillin-tazobactam MIC by method and breakpoint showed an attenuation of the mortality risk of patients with ceftriaxone non-susceptibile *Escherichia coli* and *Klebsiella pneumoniae* bloodstream infections who were treated with piperacillin-tazobactam (24). Thus, the variability of the MIC can create uncertainty in the treatment of drug-resistant pathogens (23).

Acceptance of the variability of the MIC comes at the cost of the predictive value of AST (22). As per “the 90–60” rule, if the AST result for any given antibiotic shows susceptibility, treatment with the drug is successful 90% of the time, and if the result is resistant, treatment with the drug is successful 60% of the time (22). This rule applies to immunocompetent patients infected with a single organism and treated with an intravenous drug that reaches the site of infection predictably, around 25% of inpatients (22). For the remaining 75% of patients—suffering from polymicrobial infections, receiving multiple or oral antibiotics, immunosuppressed, or with infections at sites where drug penetration is unpredictable—the predictive value of AST is lower or nonexistent (22). Precise *in vitro* measurements of antibiotic activity have the potential to form the basis of AST that is predictive of clinical outcomes and improve the diagnosis and treatment of drug-resistant infections, ultimately leading to improved patient outcomes (23).

An antibiotic dose-response curve can be generated by measuring microbial growth response to increasing concentrations of an antibiotic (25, 26). This allows for a richer estimation of *in vitro* antibiotic efficacy using metrics such as the IC_50_ (antibiotic concentration at 50% growth inhibition) and the Hill slope that are more precise than the MIC (23, 26). In this paper, we describe the use of CLSI rapidly growing NTM AST conditions to generate dose-response curves for six first-line and five novel antibiotics for MAB (14, 20). We demonstrate that antibiotic dose-response curves can be used to obtain reliable metrics for the *in vitro* activity of clinically important drugs and detect inducible resistance to clarithromycin in MAB at 5 days instead of 14.

## MATERIALS AND METHODS

### Antibiotics

Antibiotics were obtained from MedChemExpress (Monmouth Junction, New Jersey; bedaquiline, cefoxitin sodium, linezolid, SPR719, SQ109, tedizolid, and tigecycline) and Thermo Scientific Chemicals (amikacin disulfate, clarithromycin, and clofazimine). Omadacycline was provided by Paratek Pharmaceuticals (King of Prussia, Pennsylvania). Single-use antibiotic stock (concentrations in Table S1) aliquots of 10–20 μL were prepared using dimethylsulfoxide (DMSO; bedaquiline, clarithromycin, linezolid, SPR719, SQ109, tigecycline, and tedizolid) or sterile water with 0.01% Triton-X (amikacin disulfate and omadacycline) and stored at −70°C. Cefoxitin sodium (sterile water with 0.01% Triton-X) and clofazimine (DMSO) were prepared on the morning of each experiment. Antibiotics were allowed to come to room temperature prior to dispensing. Clofazimine was ultrasonicated for 10 min to facilitate dissolution.

### Strains and media

A single strain of *Mycobacterium abscessus* subspecies *abscessus* ATCC 19977 (MAB) and of *Mycobacterium peregrinum* ATCC 700686 (MPER) were used for all experiments. We used *Mycobacterium peregrinum* as it serves as a quality control strain in rapidly growing NTM AST (14). For each experiment, a frozen aliquot of both strains was subcultured onto 7H10 or 7H11 plates and incubated at 37°C for 5–10 days until there were visible colonies. The term biological replicate refers to each subculture obtained from the original strain. Similar-looking colonies were picked using a sterile loop and added to 100–200 μL of 7H9 Middlebrook media (prepared without Tween 80 or tyloxapol) in tubes pre-filled with 3 mm zirconium beads (Ops Diagnostics, Lebanon, New Jersey) and vortexed for 20–30 s. These vortexed cells were then added to 30 mL media bottles (Catalog No. 22-030595, Thermo Fisher Scientific) containing 5 mL (MAB) or 3 mL (MPER) of 7H9 media and incubated overnight in a shaking incubator (140 revolutions per minute) at 37***°*** C. Fresh cation-adjusted Mueller Hinton broth (CA-MHB) was prepared on the morning of each experimental run (as recommended by CLSI M07) by the Tufts Microbiology Media Kitchen (27).

### Experimental procedure

Antibiotic stock solutions were dispensed into the 308 non-edge wells of clear, flat-bottomed 384-well microplates using a digital drug dispenser (D300e digital dispenser; Hewlett-Packard) as described previously (28, 29). The 76 edge wells were filled with media only. For each antibiotic, the software was programmed to dispense 10 or 13 doses of specified volumes of antibiotic to obtain the desired antibiotic concentration in each well. Doses were spaced at twofold volume increments with the antibiotic concentration required to achieve 50% growth inhibition (IC_50_) at either the 5th or 6th dose (10-dose experiments) or 8th or 9th dose (13-dose experiments). Two replicates for each antibiotic were dispensed to determine technical variability. Drug wells were randomized on each plate to minimize confounding due to plate effects.

We adapted the CLSI rapidly growing NTM AST procedure for our experiments (15). For each isolate, 1.5 mL of an overnight culture was transferred from the media bottles into a microcentrifuge tube with zirconium beads and vortexed for 20–30 s. The optical density at 600 nm wavelength (OD_600_) was measured, and the culture was diluted to prepare a 0.2 OD_600_ suspension (equivalent to 0.5 McFarland Standard; confirmed by colony count) in 500–1,000 µL of CA-MHB. The final inoculum was created by adding 100 µL of 0.2 OD_600_ suspension to 20 mL of CA-MHB and 50 µL of this inoculum into each of the 308 non-edge wells.

Plates were sealed with an optically clear plate seal (AB-1170; Thermo Fisher Scientific), and OD_600_ was measured using a microplate reader (BioTek Synergy HT or Synergy H1, Agilent, Santa Clara, California). To mix the antibiotic and inoculum, plates were centrifuged for 10 s at 480 *g* in a plate centrifuge (FastGene Plate Centrifuge, Nippon Genetics Europe GmbH, Duren, Germany). They were then placed in a sealed plastic bag stacked with a maximum height of three plates and incubated at 30°C for 5 days.

### Plate reads and generation of dose-response curves

OD_600_ was measured for each plate on days 3, 4, and 5 using the optical plate reader. Plates were spun in the plate centrifuge (20–60 s at 480 *g*), and seals were changed before each plate read to eliminate condensation under the seal that could interfere with OD_600_ measurement. The plate reads were processed and dose-response curves were generated using custom scripts as detailed previously (28, 29). Printer layouts were used to derandomize plate reads to allow organization of OD_600_ reads by antibiotic. The median OD_600_ of the edge wells (OD_bkgd;_ containing only media) was subtracted from the raw OD_600_ values of non-edge wells to calculate the background-adjusted OD_600_ for all wells. The growth inhibition (*G_i_*) at any given antibiotic concentration was calculated by normalizing the bacterial growth at the corresponding concentration (OD_trt_) and normalizing it to the median OD_600_ of the untreated wells (OD_unt_), and subtracting it from 1, as shown in the equation below.


$$G_{i}=1-\;\frac{OD_{trt}}{OD_{unt}}.$$

The resulting range of growth inhibition values corresponding to increasing antibiotic concentrations were used to fit dose-response curves with a three-parameter Hill function using either the Levenberg-Marquardt algorithm or the trust-region-reflective algorithm; the results from the fit with the higher *R*^2^ value were chosen (28). The quality of each curve fit was assessed using a combination of the *R*^2^ and visual inspection of the curve. The MIC was recorded as the lowest concentration at which 100% growth inhibition was calculated, which is in contrast to MIC measurement by visual inspection used in the CLSI NTM AST broth microdilution reference method (15). The dose-response curve was used to obtain antibiotic inhibitory concentrations (IC) at 10% (IC_10_), 25% (IC_25_), 50% (IC_50_), 75% (IC_75_), 90% (IC_90_), and 95% (IC_95_) growth inhibition, respectively (30). The Hill function was used to obtain the area under the dose-response curve at 25% growth inhibition (AUC_25_), the Hill slope, and the maximum effect achievable (E_inf_). The AUC_25_ is a normalized measure of potency that allows comparison between antibiotics and reflects the amount of antibiotic required to achieve 25% growth inhibition; antibiotics with low AUC_25_ are more potent than antibiotics with high AUC_25_ (28).

### Analysis of metrics

We measured the variability of the MIC and IC values using two different methods. The first method used a range spanning one twofold concentration above and below the geometric mean of the MIC, as recommended by the CLSI (21). The second method used a range that was one geometric standard deviation above and below the geometric mean. To compare the variability between different IC measurements within and across different antibiotic-bacterial pairs, we calculated the median coefficient of variation using the ratio of the median absolute deviation (for any given IC or MIC) to the corresponding median (31). The accuracy of the MIC measurements was compared to CLSI QC ranges (MPER) or breakpoints (MAB) (14). For antibiotics not covered under CLSI guidelines (bedaquiline, omadacycline, tedizolid, tigecycline, and SPR719), we used values from studies that used CLSI rapidly growing NTM AST methodology; there were no values available for clofazimine or SQ109 (32–35). We also calculated the median for the AUC_25_, E_inf_, and Hill slope. Analyses were performed in R (version 4.5.2) and RStudio (version 2025.9.1.401) using the tidyverse (version 2.0.0) suite of packages (36–38)

## RESULTS

We performed experiments using eight biological replicates for each species (MPER and MAB). For each biological replicate, we obtained measurements from 2 technical replicates for all experimental runs (except one) with 15 replicates per antibiotic, except for clofazimine (13 replicates) and SQ109 (16 replicates). We generated 990 dose-response curves across these 66 combinations of species, antibiotics, and time points. An example of a dose-response curve and the metrics obtained from it are shown in Fig. S1. MICs were calculated for all antibiotic-bacterial pairs with the data used to generate dose-response curves and are discussed in the supplementary data and figures (Fig. S2 to S4; Table S2)

### The *R*^2^ is a reliable measure of dose-response curve quality

**Fig 1:**
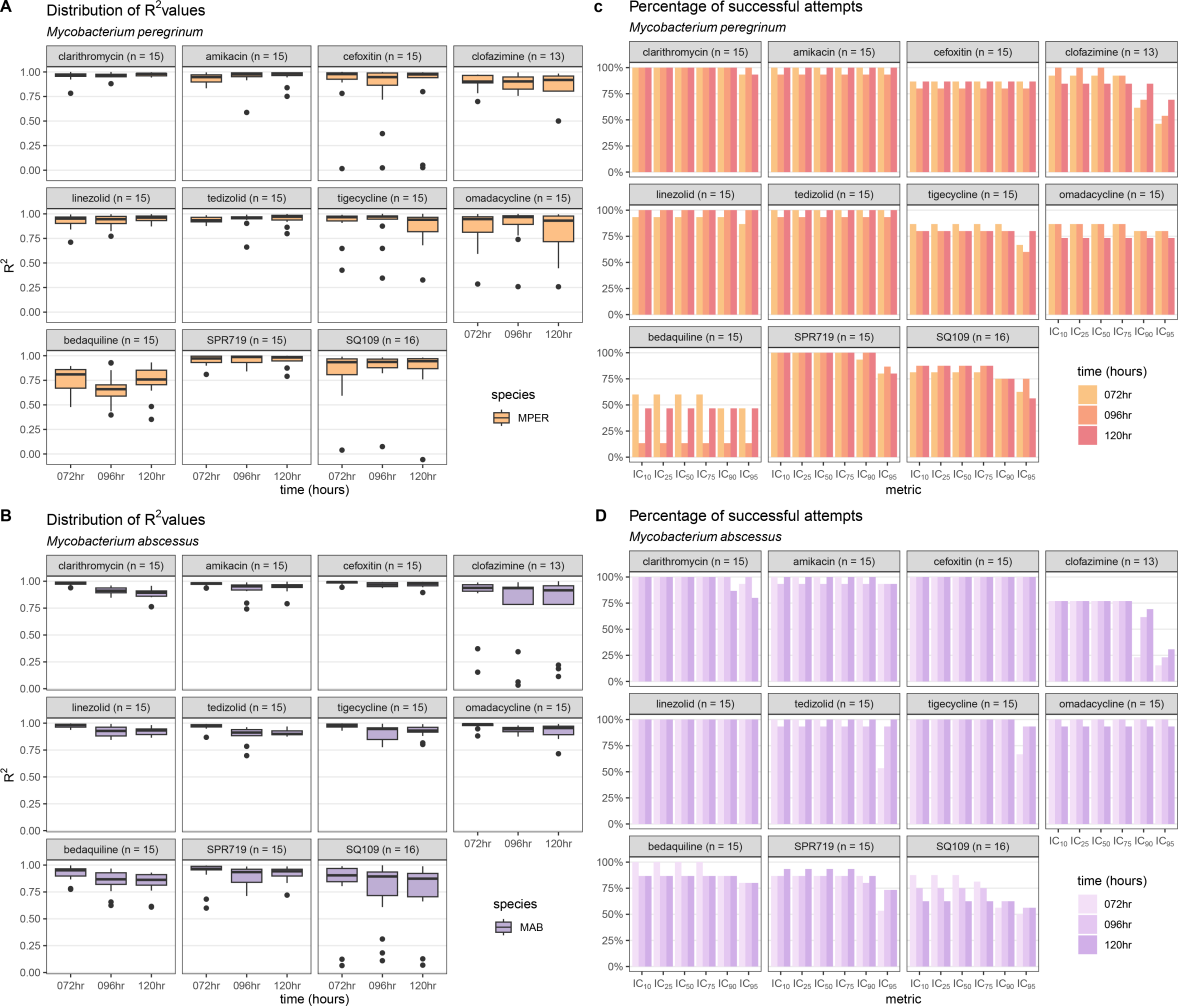
Assessment of dose-response quality. We assessed the quality of our dose-response curves using the median *R*^2^ values and the percentage of obtained inhibitory concentration (IC) metrics. *R*^2^ values measure the fit of the dose-response curve with the observed growth inhibition and range from 0 (no agreement) to 1 (complete agreement). Boxplots of the *R*^2^ of each replicate (number of attempts in parentheses) for each antibiotic for *Mycobacterium peregrinum* (**A**) and *Mycobacterium abscessus* (**B**). The percentage of IC values that could be obtained from the fitted dose-response curve for each antibiotic (number of attempts in parentheses) at each time point was calculated for *Mycobacterium peregrinum* (**C**) and *Mycobacterium abscessus* (**D**). MPER, *Mycobacterium peregrinum*; MAB, *Mycobacterium abscessus* (**B**); IC_10_, inhibitory concentration at 10% growth inhibition; IC_25_, inhibitory concentration at 25% growth inhibition; IC_50_, inhibitory concentration at 50% growth inhibition; IC_75_, inhibitory concentration at 75% growth inhibition; IC_90_, inhibitory concentration at 90% growth inhibition; IC_95_, inhibitory concentration at 95% growth inhibition; hr, hours.

The *R*^2^ (ranging between 0 and 1) compares the fitted dose-response curve (using three-parameter Hill curves) with the observed growth inhibition and was used to measure the quality of the fit. The median *R*^2^ value was greater than 0.9 for 58 of 66 antibiotic-bacterial-time-point combinations (Fig. 1A and B; Table S2). The lowest median *R*^2^ values observed were for MPER-bedaquiline (all three time points) and MAB-bedaquiline and MAB-SQ109 (96 h and 120 h).

Antibiotic-bacterial pairs with low median *R*^2^ values were likely to have a lower percentage of successful attempts at obtaining IC values (Fig. 1). For example, for MPER-bedaquiline, the median *R*^2^ values ranged from 0.66 to 0.81, with the corresponding percentage of acceptable dose-response curves ranging from 13% to 60%. In contrast, all MAB-cefoxitin replicates had acceptable dose-response curves, with median *R*^2^ values of 0.99, 0.97, and 0.98 at 72 h, 96 h, and 120 h, respectively. For both MPER and MAB, there were fewer IC_90_ and IC_95_ measurements than IC_10_–IC_75_ measurements for clofazimine, tigecycline, SPR719, and SQ109 (Fig. 1C and D). Except for clofazimine (MPER and MAB), bedaquiline (MPER), and SQ109 (MAB), the percentage of successful measurements remained consistent at each time point.

These data suggested that the *R*^2^ could be used as a proxy for quality, with dose-response curves with low *R*^2^ values being unlikely to be of acceptable quality or yield IC values. Filtering out dose-response curves with *R*^2^ values below 0.75 and that did not pass visual inspection improved the median *R*^2^ to 0.85 for all conditions (Table S2). After this filtering step, 888 dose-response curves (89.7%) were available for further analysis.

### IC values are less variable than the MIC

**Fig 2:**
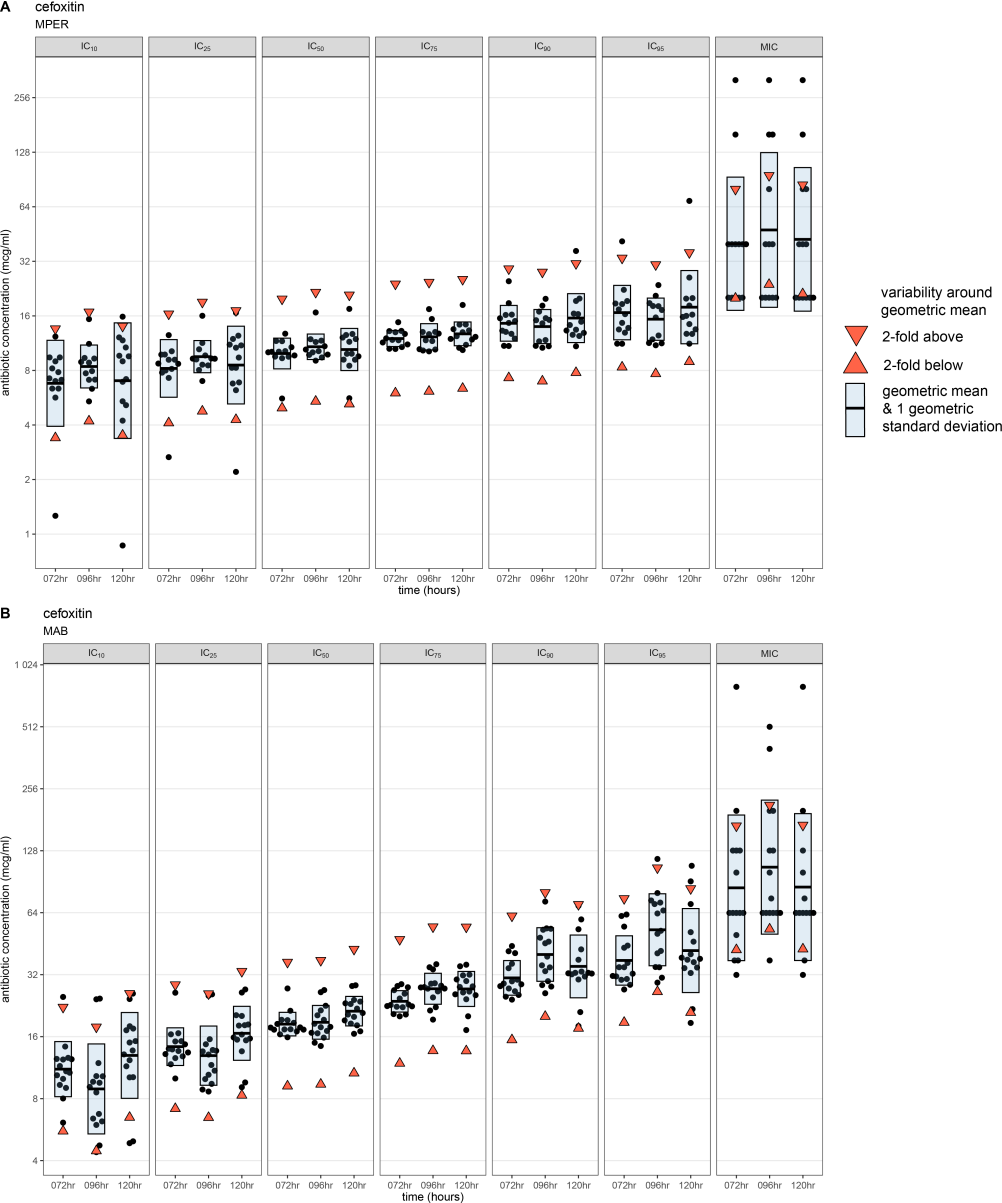
Variability of IC metrics and MIC for cefoxitin. We assessed the variability of our results using the distribution around the geometric mean of each metric. The inhibitory concentration (IC) values for each replicate and the minimum inhibitory concentrations (MICs) for cefoxitin at each time point (black points) for *Mycobacterium peregrinum* (**A**) and *Mycobacterium abscessus* (**B**), with the geometric mean represented by the horizontal bar. The upper and lower limits of ±1 twofold range around the geometric mean are represented by the downward- and upward-pointing red triangles, respectively. The blue shaded area represents 1 geometric standard deviation around the geometric mean. MPER, *Mycobacterium peregrinum*; MAB, *Mycobacterium abscessus* (**B**); IC_10_, inhibitory concentration at 10% growth inhibition; IC_25_, inhibitory concentration at 25% growth inhibition; IC_50_, inhibitory concentration at 50% growth inhibition; IC_75_, inhibitory concentration at 75% growth inhibition; IC_90_, inhibitory concentration at 90% growth inhibition; IC_95_, inhibitory concentration at 95% growth inhibition; hr, hours.

We determined the variability of IC metrics using either (i) ±1 twofold concentration or (ii) 1 geometric standard deviation around the geometric mean of all replicates for any given metric (Table S2). We posited that the more precise the IC value or MIC, the narrower the spread of the geometric standard deviation around the geometric mean. Using cefoxitin as an example (Fig. 2), only 1–2 values were outside the twofold range (limits shown with red triangles) of the geometric mean (horizontal black line) for IC_10_, IC_25_, and IC_95_ for MPER and MAB. For all IC values, the range of the geometric mean and 1 geometric standard deviation (blue rectangle) was less than the twofold doubling range around the corresponding geometric mean (Fig. 2). In contrast, for the MIC, the range of the geometric mean and geometric standard deviation was greater than the fourfold range of the geometric mean for both MAB and MPER.

The reproducibility of IC metrics varied across antibiotic-bacterial pairs (Fig. 2; Fig. S4 to S6). Except for MPER-bedaquiline, MAB-tigecycline, MAB-SPR719, and MAB-SQ109, most replicates for IC_50_ and IC_75_ were within ± twofold concentration (range between red triangles) of the geometric mean (horizontal black line) and the spread of 1 geometric standard deviation around the geometric mean (blue shaded area) was less than the twofold concentration range around the geometric mean (range between red triangles). Across antibiotic-bacterial pairs and time points, we noted greater variability around the geometric mean for the IC_10,_ IC_25_, IC_90_, and IC_95_ than for the IC_50_ and IC_75_.

### The IC_50_ and IC_75_ values are the least variable IC values

Because the median IC and MIC values approximated the geometric mean (Table S2), we used the median coefficient of variation (the non-parametric equivalent of the coefficient of variation) to compare the variability across different IC values and different antibiotic-bacterial pairs. A median absolute deviation equal to the median IC would be equivalent to a ±1 twofold concentration around the median IC and result in a median coefficient of variation of 1. We therefore defined a median coefficient of variation of 1 or lower as an acceptable limit of variability.

**Fig 3:**
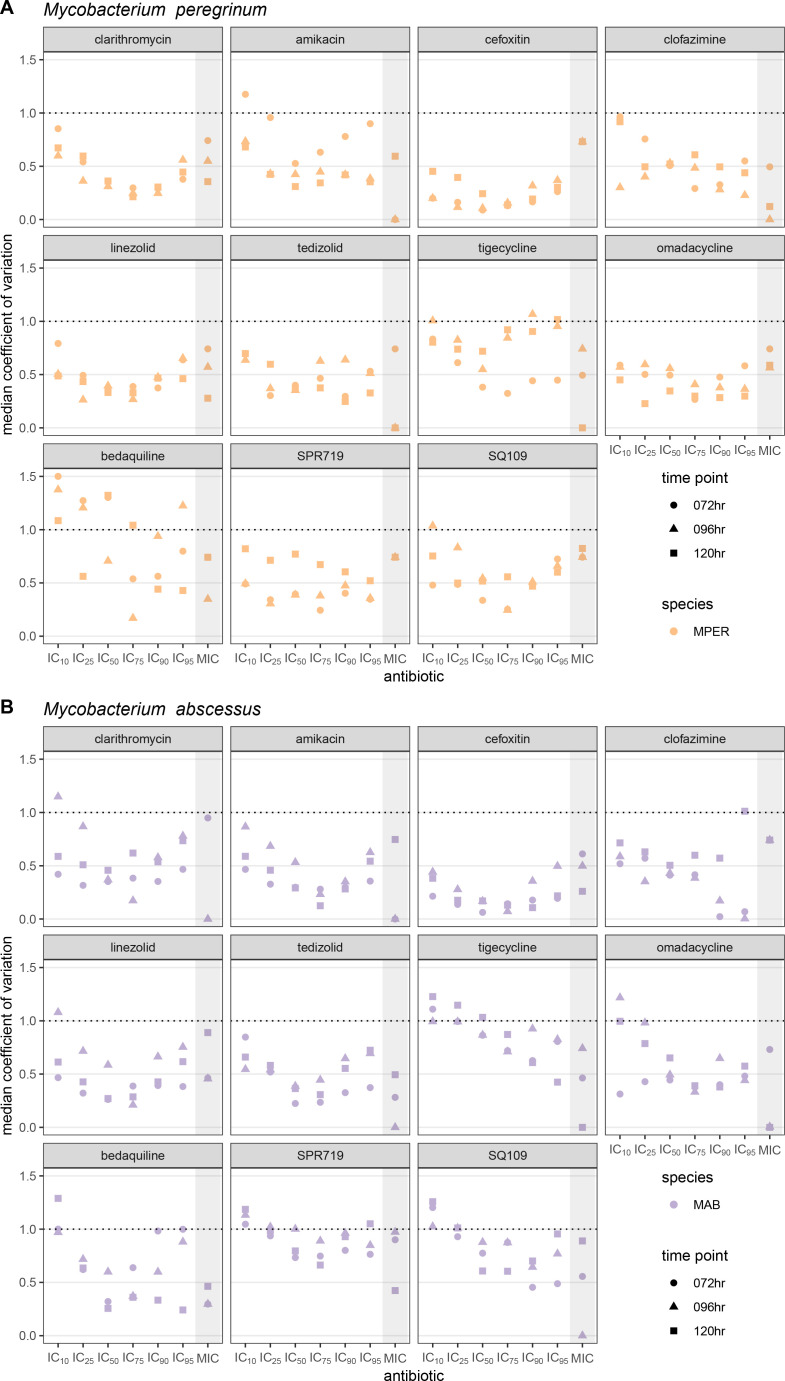
Comparison of the median coefficient of variation across IC and MIC values, time points, and antibiotics. The median coefficient of variation is the nonparametric equivalent of the coefficient of variation and allows comparison of variability across multiple dimensions of strain, antibiotic, and IC values. It was calculated for *Mycobacterium peregrinum* (**A**) and *Mycobacterium abscessus* (**B**). We used a value of 1 or lower as an acceptable amount of variability, as it represents the value at which the median standard deviation is equal to the median and would be equivalent to ±1 twofold range around the median. MPER, *Mycobacterium peregrinum*; MAB, *Mycobacterium abscessus* (**B**); hr, hours.

Except for MPER-bedaquiline, the median coefficient of variation was less than one for most IC values for all antibiotic-bacterial pairs; the IC_10_ was the IC value for which the median coefficient of variation most frequently exceeded 1 (Fig. 3). We found that the median coefficient of variation was lowest for the IC_50_ and IC_75_ than for other IC values at any given time point, demonstrating a U-shape (Fig. 3). This U-shaped pattern was best seen with cefoxitin and was present across most antibiotic-bacterial pairs (Fig. 3). Exceptions to this U-shaped pattern included clofazimine and tigecycline (both MPER and MAB) and bedaquiline-MPER, for which there were both lower median *R*^2^ values and lower percentage of successful attempts at obtaining IC values (Fig. 1; Table S2). Antibiotic-bacterial pairs with lower median or wider spread of *R*^2^ values tended to have higher median coefficients of variation across all IC values, while preserving the U-shaped pattern to varying degrees, as seen with SPR719 and SQ109 (Fig. 3).

### Dose-response metrics can identify inducible resistance to clarithromycin

**Fig 4:**
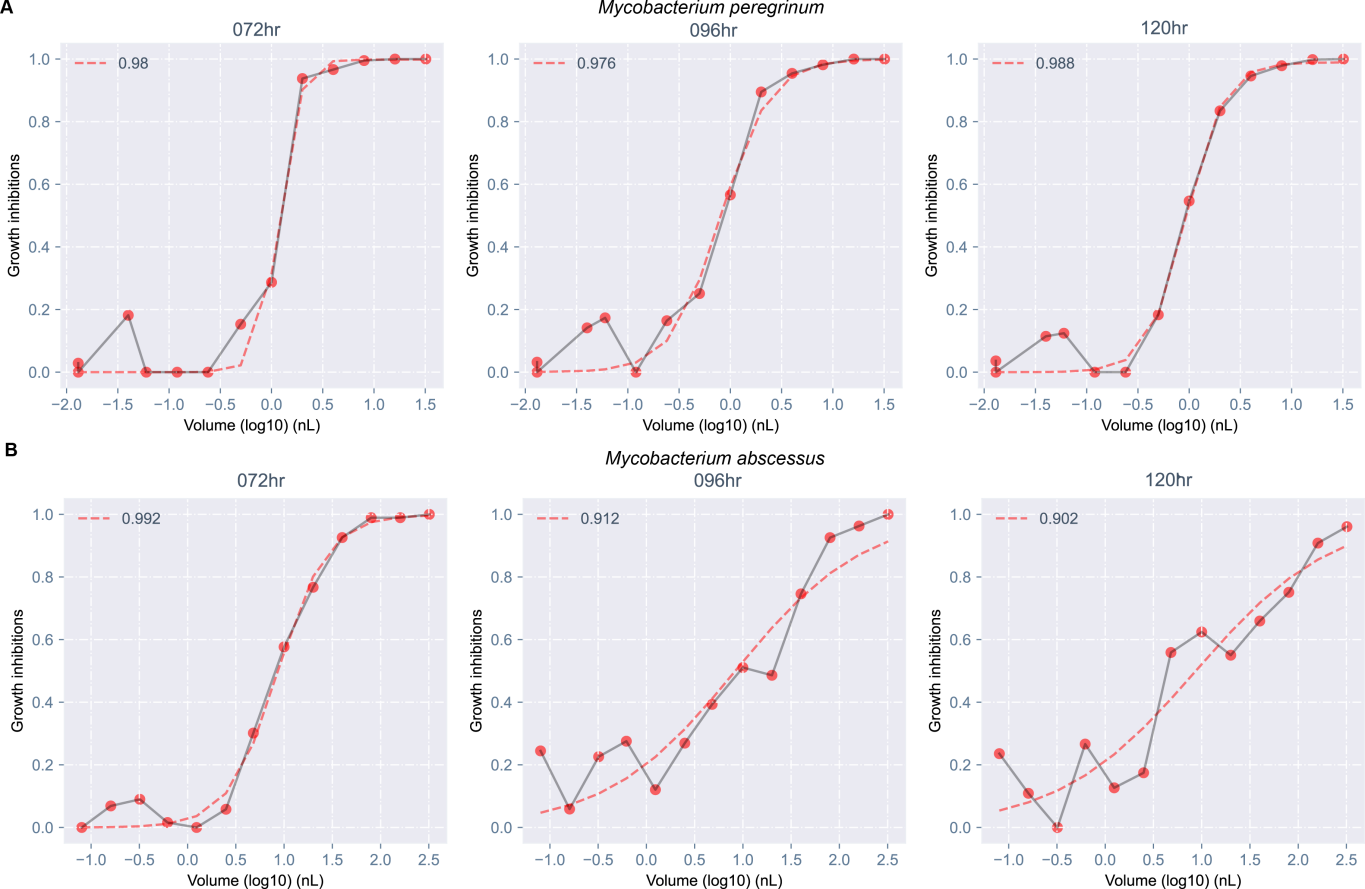
Temporal changes in dose-response curves consistent with inducible resistance to clarithromycin in *Mycobacterium abscessus*. Dose-response curves for clarithromycin showed no change in shape across time for *Mycobacterium peregrinum* (**A**). However, changes in shape were seen for *Mycobacterium abscessus* with decreased growth inhibition at each successive time point (three concentrations at 100% growth inhibition at 72 h, one concentration at 96 h, and none at 120 h), as well as flattening of the curve (decrease in slope) (**B**). The red points show calculated growth inhibitions at each dispensed concentration and are connected by a gray line. The red dashed line is the dose-response curve fitted with a three-parameter Hill curve. The number in the top left corner of each plot is the *R*^2^, which measures the quality of the fitted curve. hr, hours.

**Fig 5:**
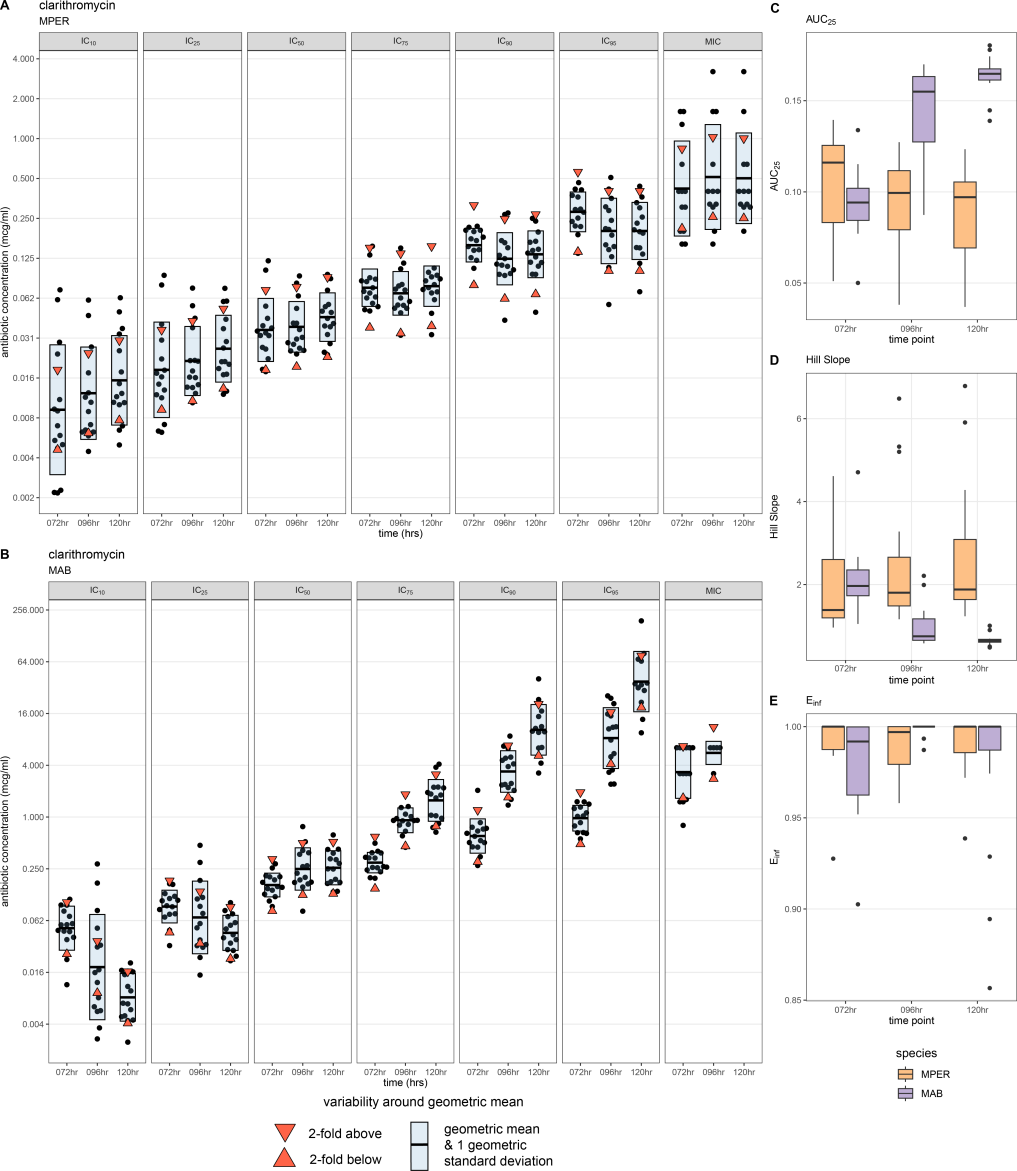
Dose-response metrics show inducible resistance to clarithromycin in *Mycobacterium abscessus*. Changes in the shape of the dose-response curve are matched by changes in dose-response curve metrics. The IC values for each replicate and the MICs for cefoxitin at each time point (black points) for *Mycobacterium peregrinum* (**A**) and *Mycobacterium abscessus* (**B**), with the geometric mean represented by the horizontal bar. The upper and lower limits of ±1 twofold range around the geometric mean are represented by the downward- and upward-pointing red triangles, respectively. The blue shaded area is 1 geometric standard deviation around the geometric mean. Metrics from the dose-response curve can also show evidence of inducible resistance as seen with boxplots of the area under the curve at 25% growth inhibition (AUC_25_, **C**) and the Hill Slope (**D**). These are not seen with the E_inf_ (maximum effect at infinite concentration, **E** ). MPER, *Mycobacterium peregrinum*; MAB, *Mycobacterium abscessu*s (**B**); IC_10_, inhibitory concentration at 10% growth inhibition; IC_25_, inhibitory concentration at 25% growth inhibition; IC_50_, inhibitory concentration at 50% growth inhibition; IC_75_, inhibitory concentration at 75% growth inhibition; IC_90_, inhibitory concentration at 90% growth inhibition; IC_95_, inhibitory concentration at 95% growth inhibition; hr, hours.

The shape of MAB-clarithromycin (Fig. 4B) dose-response curves (dashed lines) changed over time; these changes were absent in MPER-clarithromycin (Fig. 4A). We obtained fewer MIC readings at 96 h than at 72 h, and none at 120 h (Fig. 5B). On plotting the IC values, we noted the IC_50_ value remained unchanged across all time points and the IC_75_, IC_90_, and IC_95_ values increased with successive time points (Fig. 5B). Similarly, the AUC_25_, a measure of antibiotic potency, increased over time (Fig. 5C), consistent with increasing concentration of antibiotic necessary to achieve the same amount of growth inhibition at successive time points. On the other hand, the Hill slope decreased over time (Fig. 5D), suggesting that achieving complete growth inhibition would require a higher concentration of the antibiotic at later time points. All these metrics remained unchanged over time in MPER (Fig. 5A and C through E). We believe that these findings are likely explained by inducible resistance mediated by the *erm*(41) gene in the MAB ATCC type strain used in our experiments; this finding was not observed in MPER, which is not known to harbor any of the *erm* genes responsible for inducible resistance to clarithromycin (15).

## DISCUSSION

In this study, we demonstrate that antibiotic activity can be measured using dose-response curves, yielding metrics that are less variable than the MIC and that can identify inducible resistance at 5 days. The consequences of flaws in MIC-based AST are best seen with MAB, where multiple antibiotics with significant toxicity are used for months to years with limited or no clinical evidence of their utility; the absence of reliable and predictive metrics has hindered the search for effective and safe treatment for patients infected with this highly drug-resistant pathogen (1, 5, 7, 8, 11). This unreliability of MIC-based AST in antibiotic selection is seen with other NTMs. CLSI guidelines explicitly recommend against testing ethambutol and rifampin for members of *Mycobacterium avium* complex, and isoniazid and ethambutol for *M. kansasii*, even though treatment guidelines recommend these drugs as first-line for these infections (3, 14, 15).

We demonstrate that AST performed using dose-response curves may have two potential advantages over MIC-based AST methods. First, we show that the IC_50_ and IC_75_ allow precise measurement of antibiotic activity over an 8,000-fold range in concentration, in contrast to existing AST methods that measure the MIC over a narrower range (32- to 500-fold for all drugs except imipenem on the RAPMYCO2 Sensititre™ panel made by Thermo Fisher Scientific). Previous work has demonstrated challenges in obtaining reproducible results with NTM AST, even when testing is performed by reference laboratories (39–42). For AST performed using broth microdilution using three clinical strains of MAB, essential agreement of MIC results was 47.2%–100% for clarithromycin, and 97.2%–100% for both amikacin and cefoxitin (42). When including results from additional antibiotics and three strains of *M. fortuitum* and four strains of *M. chelonae*, there was ≥90% essential agreement of MIC for only 70% of antibiotic-bacteria combinations. We show that the variability of the IC_50_ and IC_75_ (measured using the geometric standard deviation) for clarithromycin, amikacin, cefoxitin, linezolid, tedizolid, and omadacycline is less than the twofold range around the geometric mean required for acceptable reproducibility of MIC testing. More precise measurement of antibiotic activity has the potential to improve many aspects of the diagnosis and treatment of drug-resistant pathogens, as discussed by Brennan-Krohn et al (23). Our findings are especially relevant to MAB infections, where AST has limited use in antibiotic selection, and antibiotic toxicity is common and frequently leads to treatment cessation (1, 3, 5, 8, 11).

Second, we identify time-dependent changes in dose-response curve metrics that enable the identification of inducible clarithromycin resistance with only 5 days of incubation. Inducible resistance could be framed as the need for a progressive increase in antibiotic concentration to achieve the same amount of growth inhibition with each successive time point and is seen in the “flattening” of the dose-response curve, which raises IC values, increases the AUC_25_, and reduces the Hill slope. We hypothesize that the effects of inducible resistance on growth inhibition are first visible at the “top of the curve” and thus seen with increases in the higher IC values (IC_75_ and above) before they are seen at lower IC values (IC_50_ and below). It is possible that if we extended incubation to 7 or 10 days, we would start to see changes in the IC_50_. Existing MAB AST can identify inducible resistance to clarithromycin in only ~60% of isolates after 5 days of incubation, necessitating the use of either extended incubation to 14 days or molecular assays (*erm*(41) sequencing or line probe assays), which are usually only available at reference laboratories (43). We propose that an increase in the IC_75_, IC_90_, IC_95_, and AUC_25_, and a decrease in the Hill slope between days 3 and 5, could serve as a marker of inducible resistance and obviate the need for extended 14-day incubation or use of molecular methods to detect inducible clarithromycin resistance in MAB. This could allow the faster identification of inducible resistance and reduce delays in treatment initiation.

Our work has many strengths that make it clinically relevant. We used CLSI standards for antibiotic stock preparation and storage (27). We performed experiments starting with an inoculum equivalent to 0.5 McFarland standard, used freshly prepared cefoxitin and CA-MHB with each experimental run, and incubated plates at 30°C for 3–5 days, all steps recommended by CLSI (15, 21). Our previous work that used conditions that differed (higher OD_600_ of starting inoculum, 7H9 culture medium, and incubation at 37°C for 2 days) from those recommended by the CLSI was characterized by challenges in obtaining consistent dose-response curves due to the inability to obtain complete growth inhibition and high levels of variability for some drugs and drug combinations (29). We tested drugs that are recommended or appear promising for the treatment of MAB (1, 3, 32, 33, 35). *R*^2^ values served as a good measure of the quality of dose-response curves and might be incorporated into clinical microbiology quality control methods for dose-response curve AST. We were able to obtain results for omadacycline, which has been challenging with conventional AST due to trailing endpoints (34, 44). We quantified the variability between different IC and MIC values, time points, and antibiotics. By incorporating dose-response curve metrics (AUC_25_ and the Hill slope), we compared antibiotic potency across species, drugs, and time points, a feature not currently possible with MICs or IC values.

Limitations of our work revolved around technical challenges. Drug-specific variability in IC and MIC values, lower median *R*^2^ values, and a low percentage of successful attempts were likely due to one or more of a combination of antibiotic insolubility (clofazimine), the use of high antibiotic stock concentrations (SQ109), and low dispense volumes (bedaquiline and tigecycline), suggesting the need for optimization of antibiotic preparation for future experiments. The dose-response curves for some antibiotics might be better fit with models other than the three-parameter Hill curve. Our threshold of 0.75 for an acceptable *R*^2^ was chosen based on our previous work, which used a threshold of 0.70, but it is possible that our results could differ with higher or lower thresholds (29). While we replaced the optically clear plate seal at each time point before obtaining OD_600_ values, condensation under the seal due to temperature differences between the incubator, biosafety cabinet, and ambient air may have introduced error in OD_600_ measurements. Experiments were performed using 50 µL of inoculum per well in a 384-well plate, unlike CLSI rapidly growing NTM testing, which uses 100 µL per well in a 96-well plate. The MIC and IC values we obtained were higher than the ranges expected using CLSI testing, likely because the optical plate reader is more sensitive than the unaided eye in measuring bacterial growth, although errors in inoculum preparation cannot be ruled out. We excluded nearly 1/3 of MIC measurements because we could not obtain 100% growth inhibition, suggesting that new susceptibility breakpoints based on IC values will need to be established for each antibiotic-bacterial pair.

Our results are not yet generalizable to clinical isolates as we used only two type strains for our experiments, both of which have smooth morphology. The heterogeneity of MAB is reflected in its three subspecies, rough and smooth morphologies, and variability in antibiotic responses driven by genetic background (45, 46). Validation of this work in clinical isolates from all three subspecies, with rough and smooth morphologies, diverse resistance mechanisms, and a range of MIC values, is needed. Rough morphology isolates could pose a particular challenge as they tend to clump, and optical densities might not be measured accurately. This could lead to variability in dose-response curves that might not be seen with smooth isolates. Dose-response curve features of inducible clarithromycin resistance might not be present or be delayed in clinical isolates due to strain-specific heterogeneity of the WhiB7 regulatory network that controls the expression of *erm*(41) (47–49). Finally, even if reliable dose-response curves can be obtained from clinical isolates, the impact of these metrics on treatment outcomes will need to be demonstrated in animal and clinical studies.

In 1992, Soothill et al. used *Pseudomonas aeruginosa* and gentamicin dissolved in solid agar plates at 12 different concentrations and demonstrated that the IC_50_ was significantly less variable than the MIC and allowed strain-specific measurements of antibiotic activity (26). Using CLSI conditions for rapidly growing NTM AST, 384-well plates, and a digital drug dispenser, we redemonstrate Soothill et al.’s findings using MAB more than 30 years later. Our results indicate that measuring antibiotic activity against MAB using dose-response curves can overcome the limitations of the MIC while allowing the rapid identification of inducible resistance to clarithromycin. These findings have the potential to improve MAB AST and lead to diagnostic and therapeutic advances that ultimately improve outcomes for patients infected with this “antibiotic nightmare” (2).

## AFTER EPUB

*[This article was published on 6 April 2026 with an error in Fig. 3. The figure was corrected in the current version, posted on 7 April 2026.]*

## SUPPLEMENTAL MATERIAL

Fig. S1 aac.01876-25-s0001.pdf Doseresponse curve example.

Fig. S2 aac.01876-25-s0002.pdf Percentage of successful MIC measurements.

Fig. S3 aac.01876-25-s0003.pdf MIC measurements of all 11 antibiotics.

Fig. S4 aac.01876-25-s0004.pdf MIC median coefficient of variation.

Fig. S5 aac.01876-25-s0005.pdf IC and MIC values amikacin clofazimine.

Fig. S6 aac.01876-25-s0006.pdf IC and MIC values linezolid, tedizolid, tigecycline, and omadacycline.

Fig. S7 aac.01876-25-s0007.pdf IC and MIC values bedaquiline, SPR719, and SQ109.

Fig. S8 aac.01876-25-s0008.pdf Hill curve metrics.

Supplemental material aac.01876-25-s0009.docx Supplemental figure legends.

Supplemental tables aac.01876-25-s0010.xlsx Tables S1 and S2.

ASM does not own the copyrights to Supplemental Material that may be linked to, or accessed through, an article. The authors have granted ASM a non-exclusive, world-wide license to publish the Supplemental Material files. Please contact the corresponding author directly for reuse.
