# Supplementary figures and images for: Antibiotic dose-response curves can measure antibiotic activity against Mycobacterium abscessus and Mycobacterium peregrinum
Source: Antimicrob Agents Chemother. 2026 Apr 6;70(5):e01876-25. doi: 10.1128/aac.01876-25 (PMC13148060; doi:10.1128/aac.01876-25)

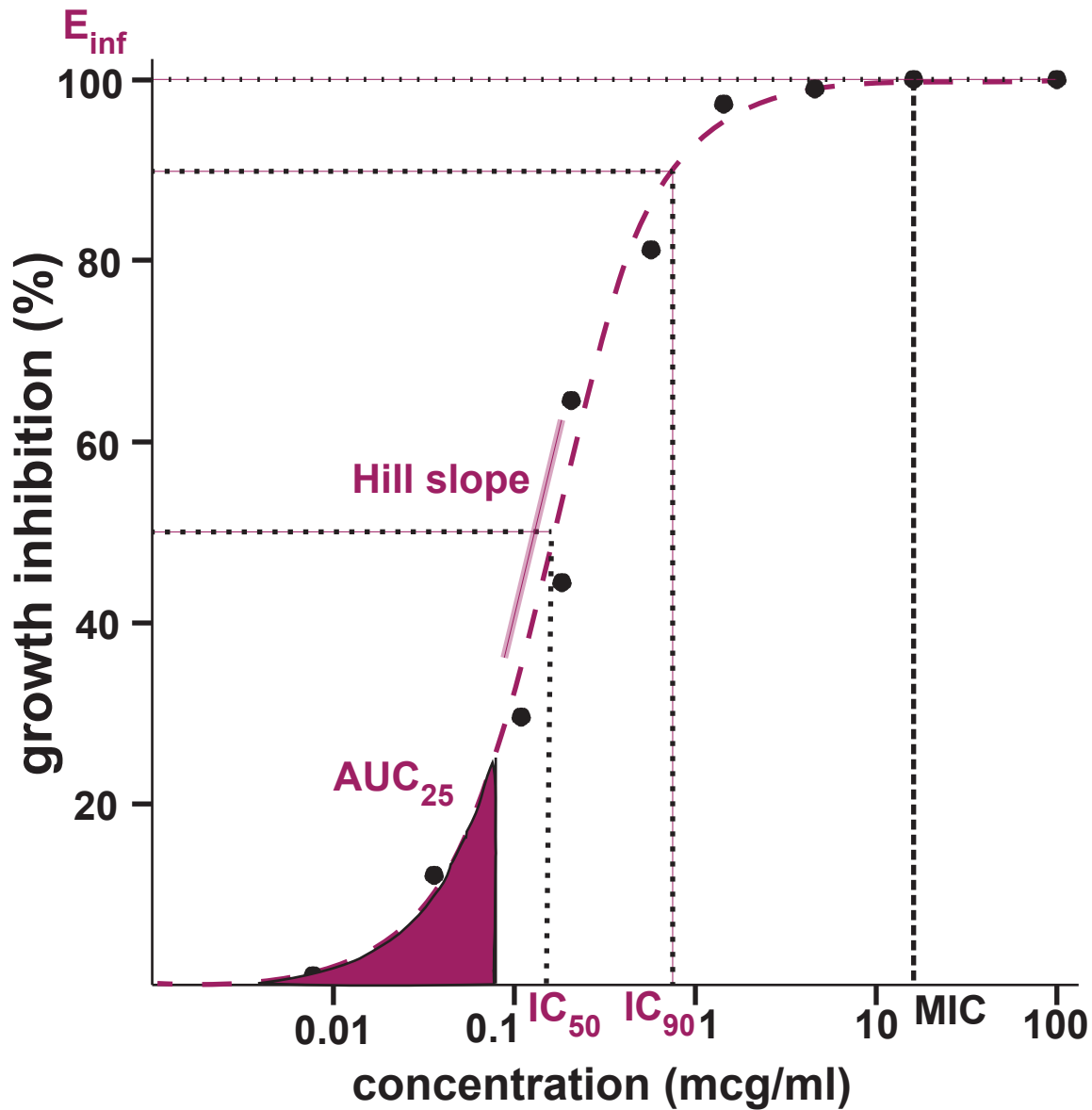

Supplement: Fig. S1 — Doseresponse curve example. [file aac.01876-25-s0001.pdf]

**A***Mycobacterium peregrinum*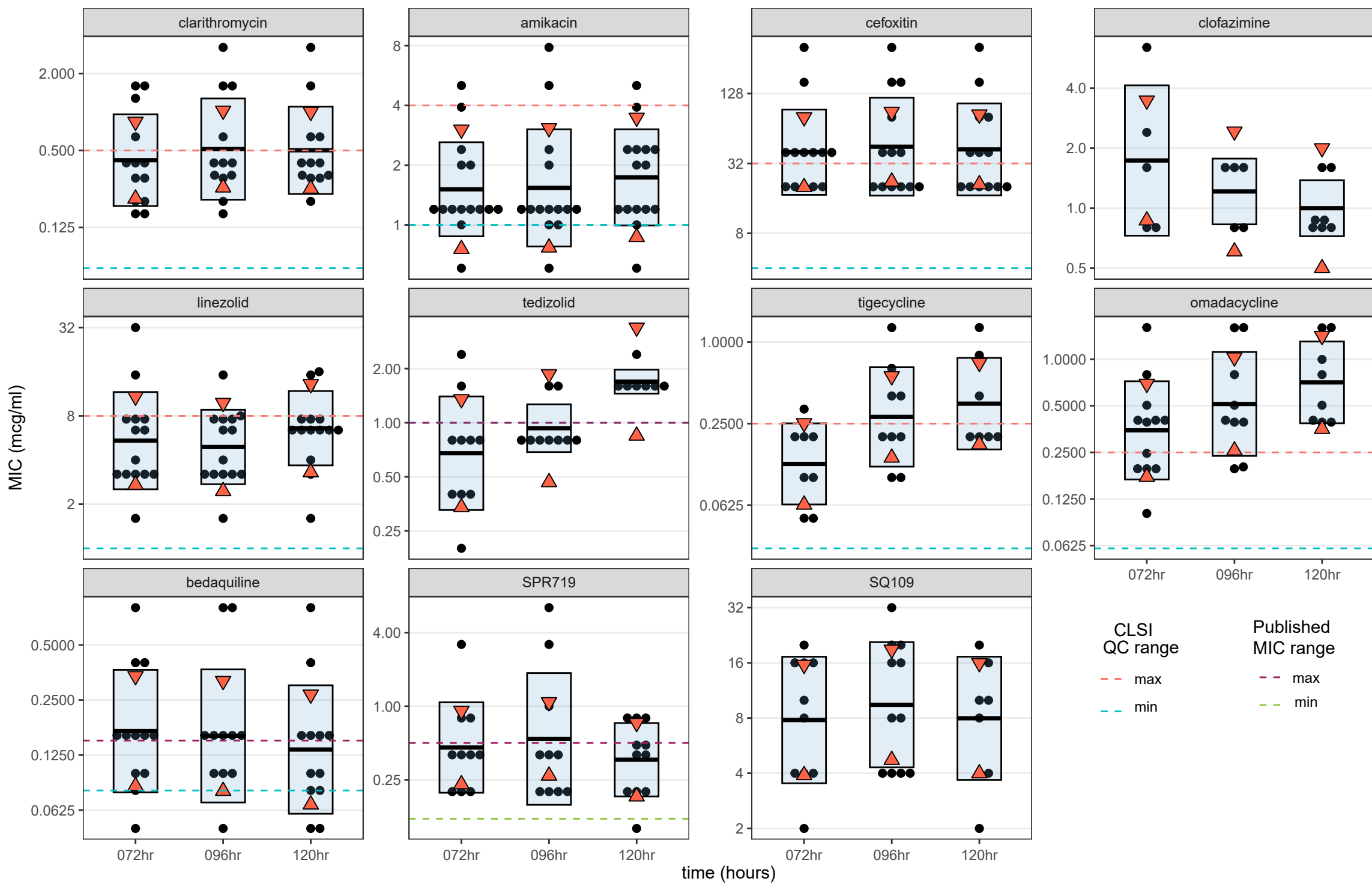**B***Mycobacterium abscessus*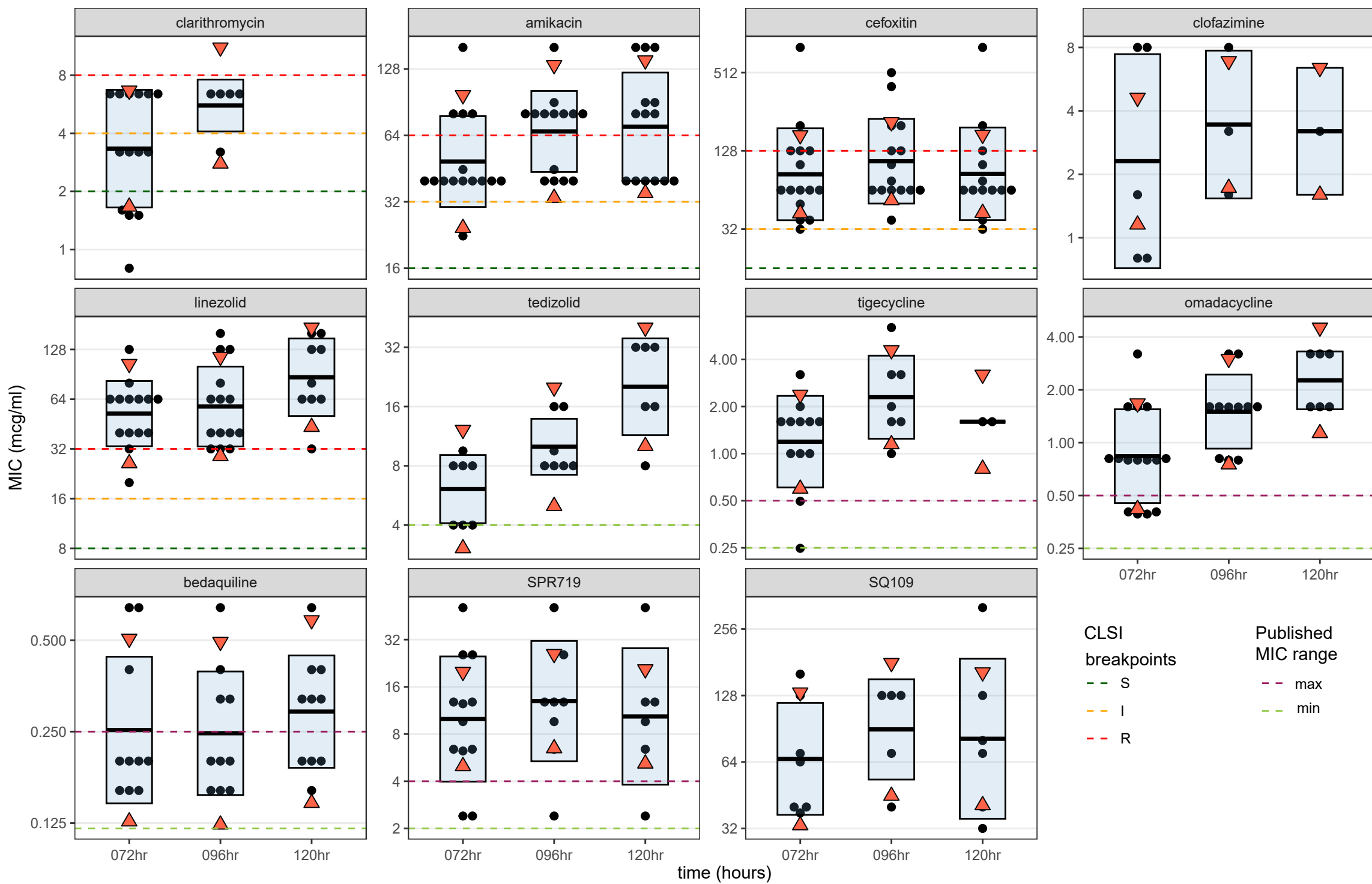

Supplement: Fig. S3 — MIC measurements of all 11 antibiotics. [file aac.01876-25-s0003.pdf]

**C** Median coefficient of variation of MIC measurements

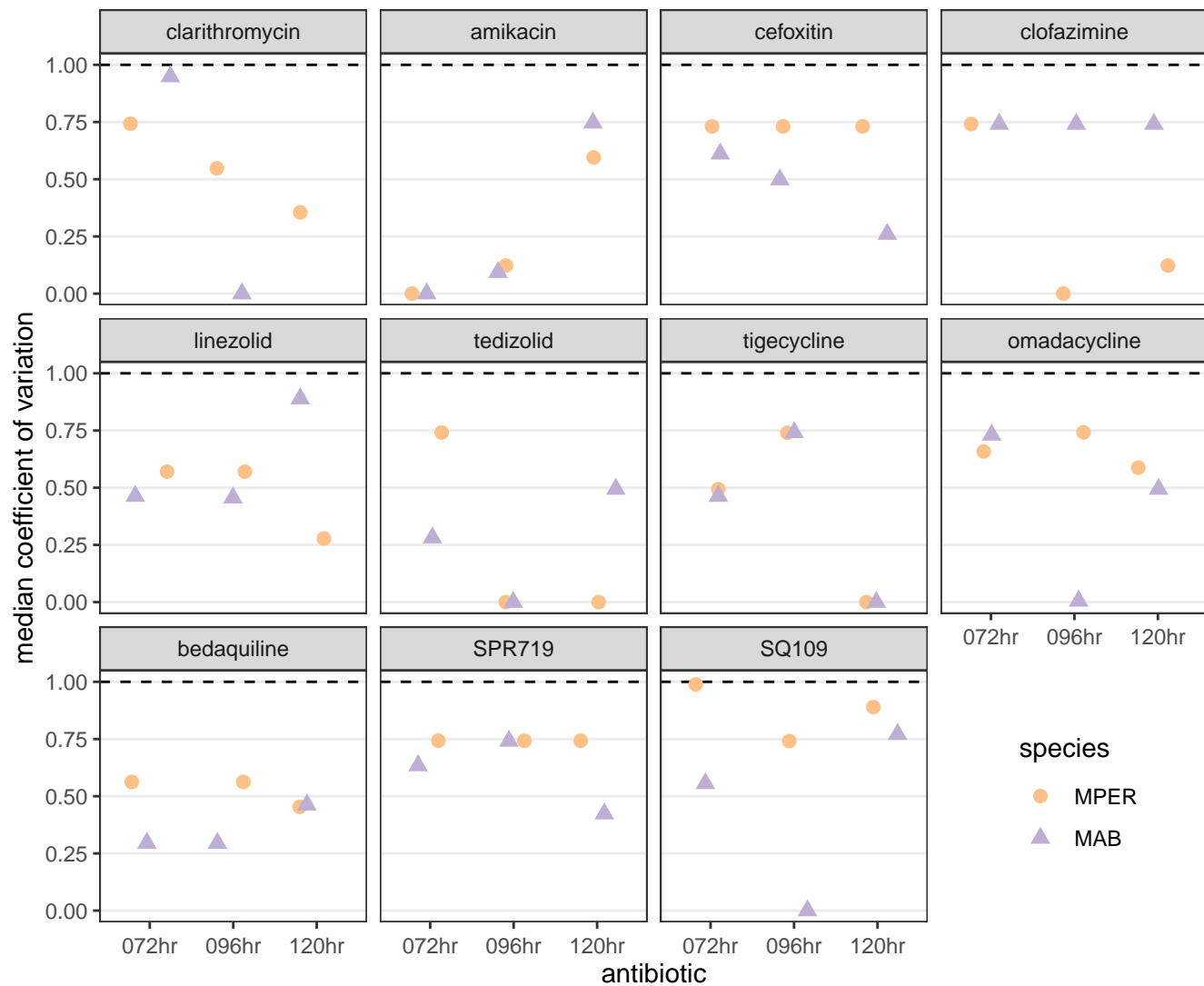

Supplement: Fig. S4 — MIC median coefficient of variation. [file aac.01876-25-s0004.pdf]

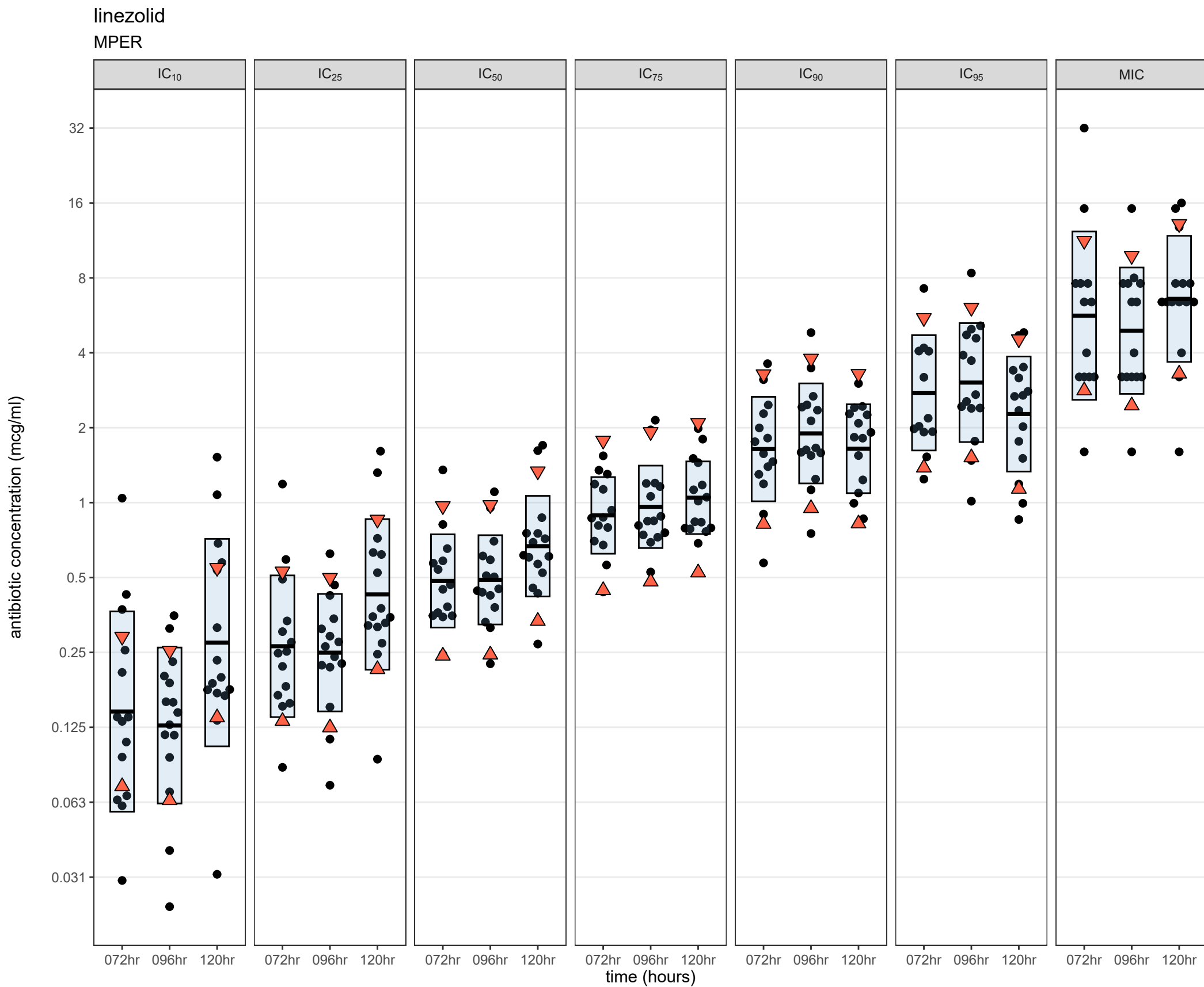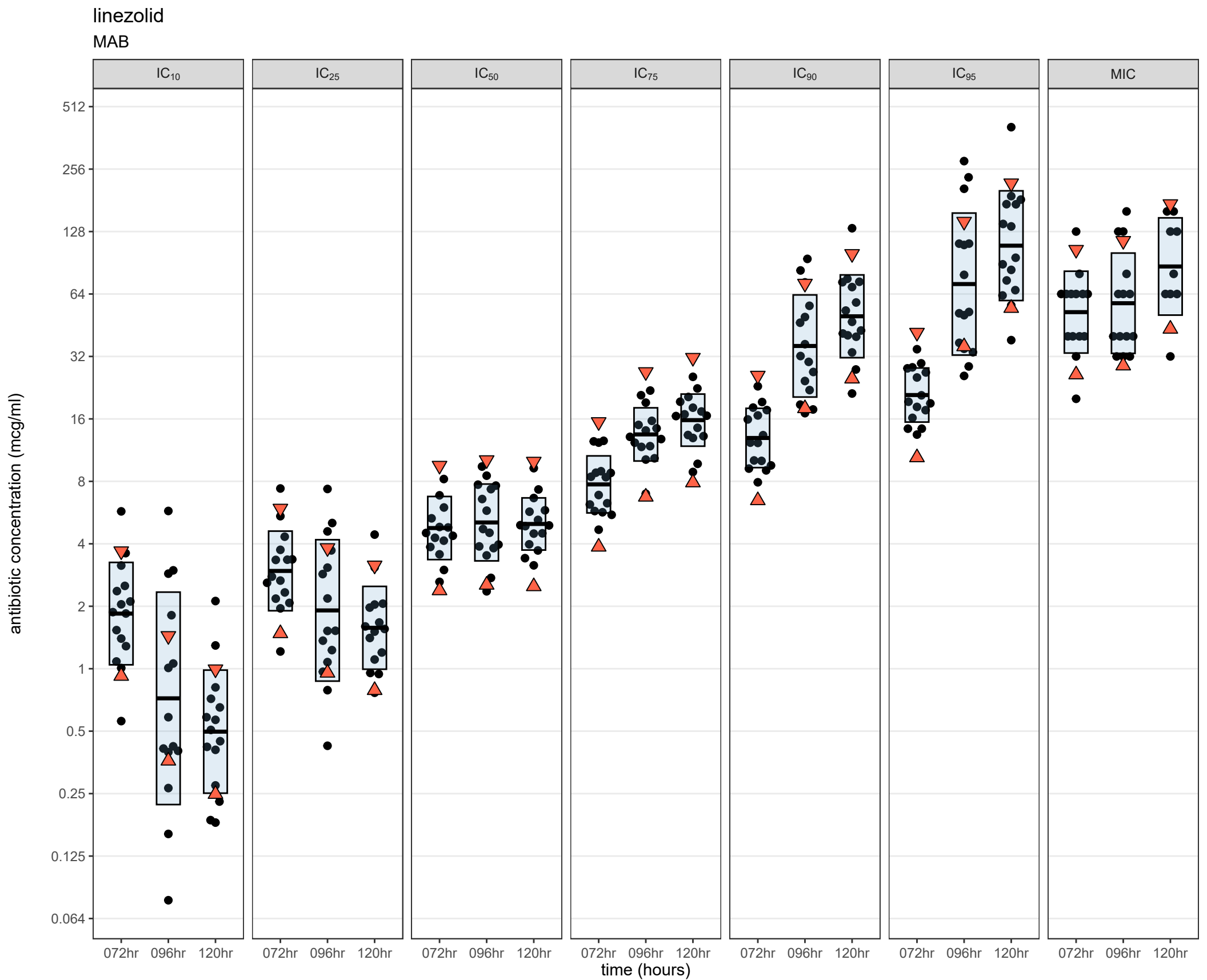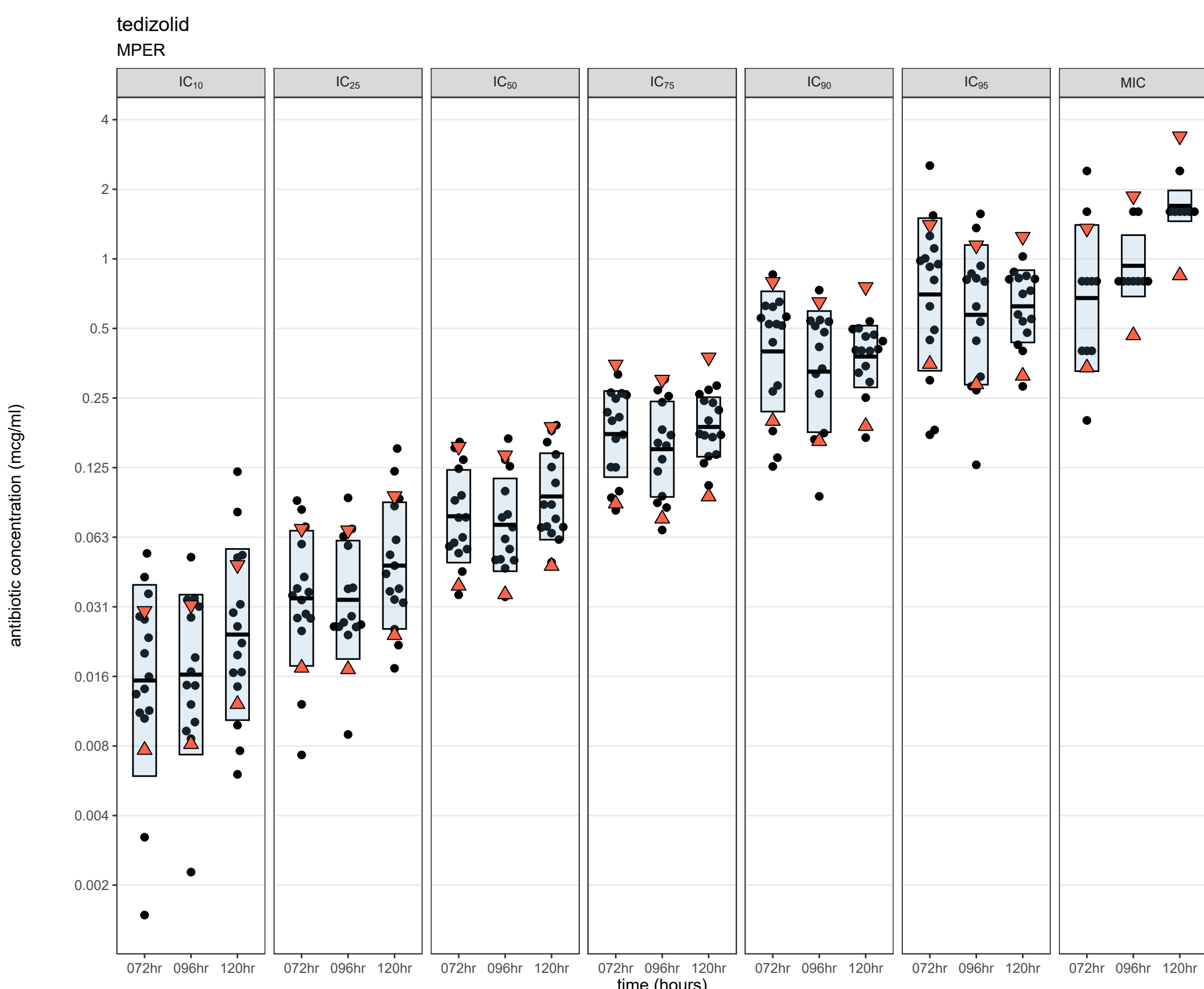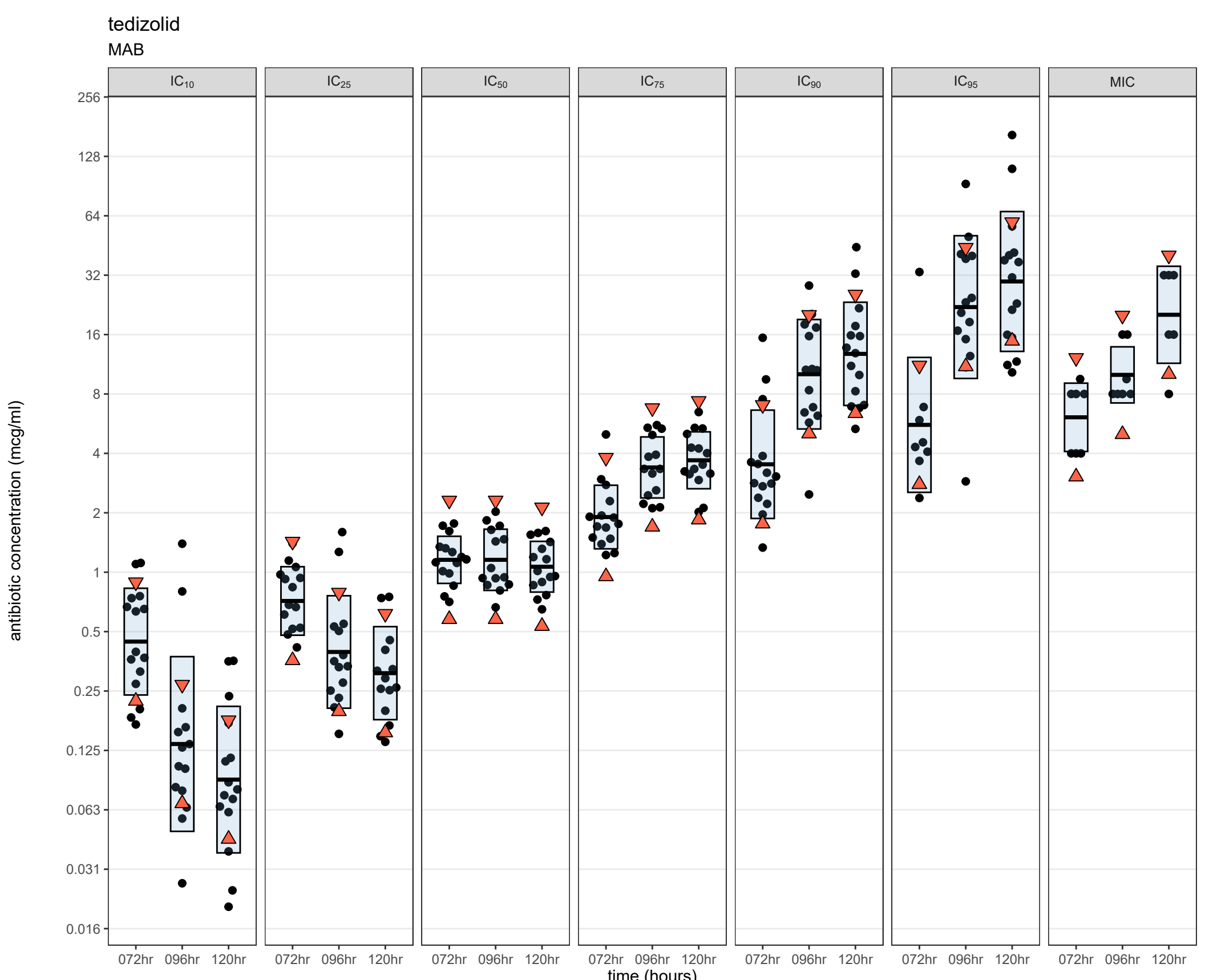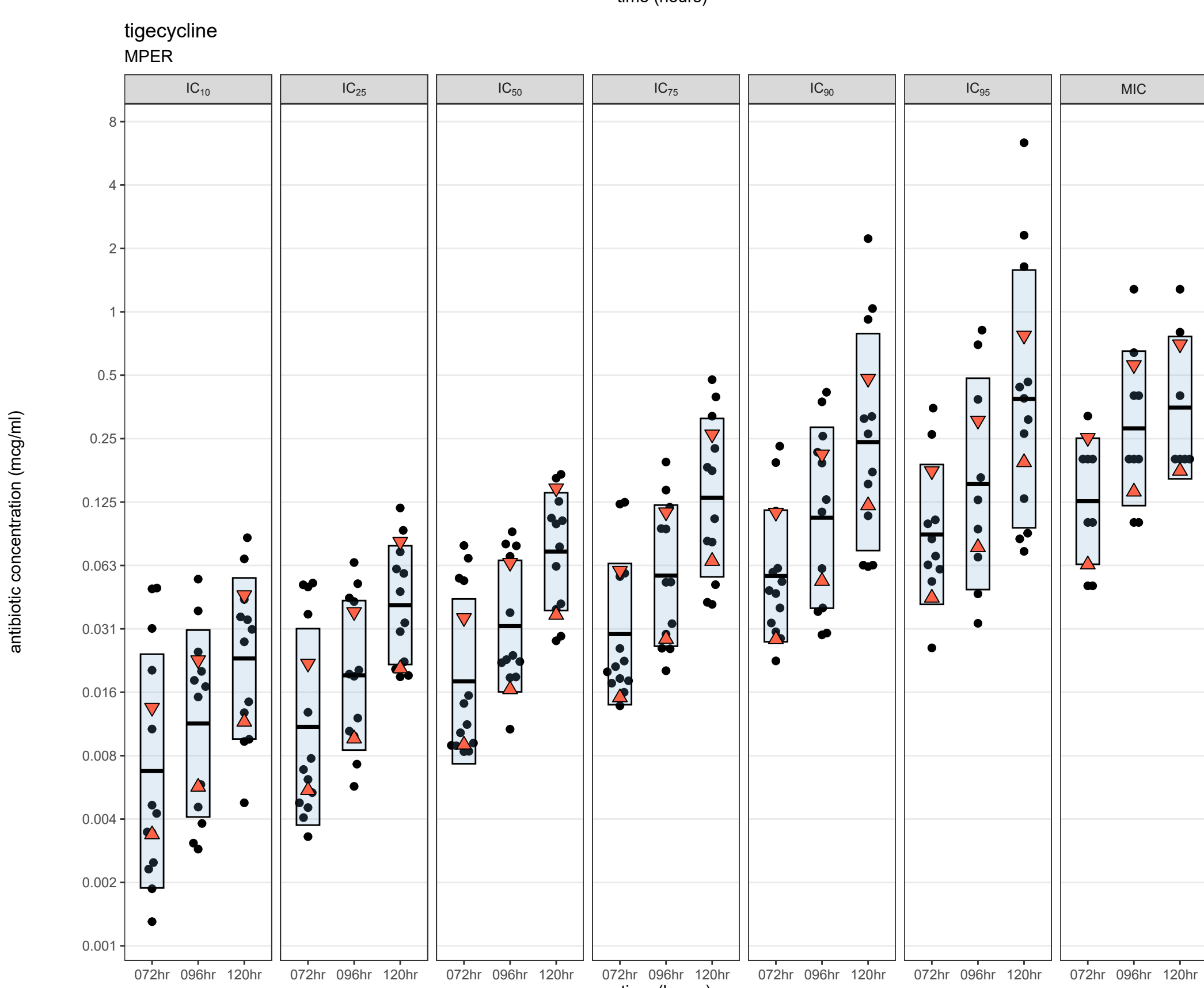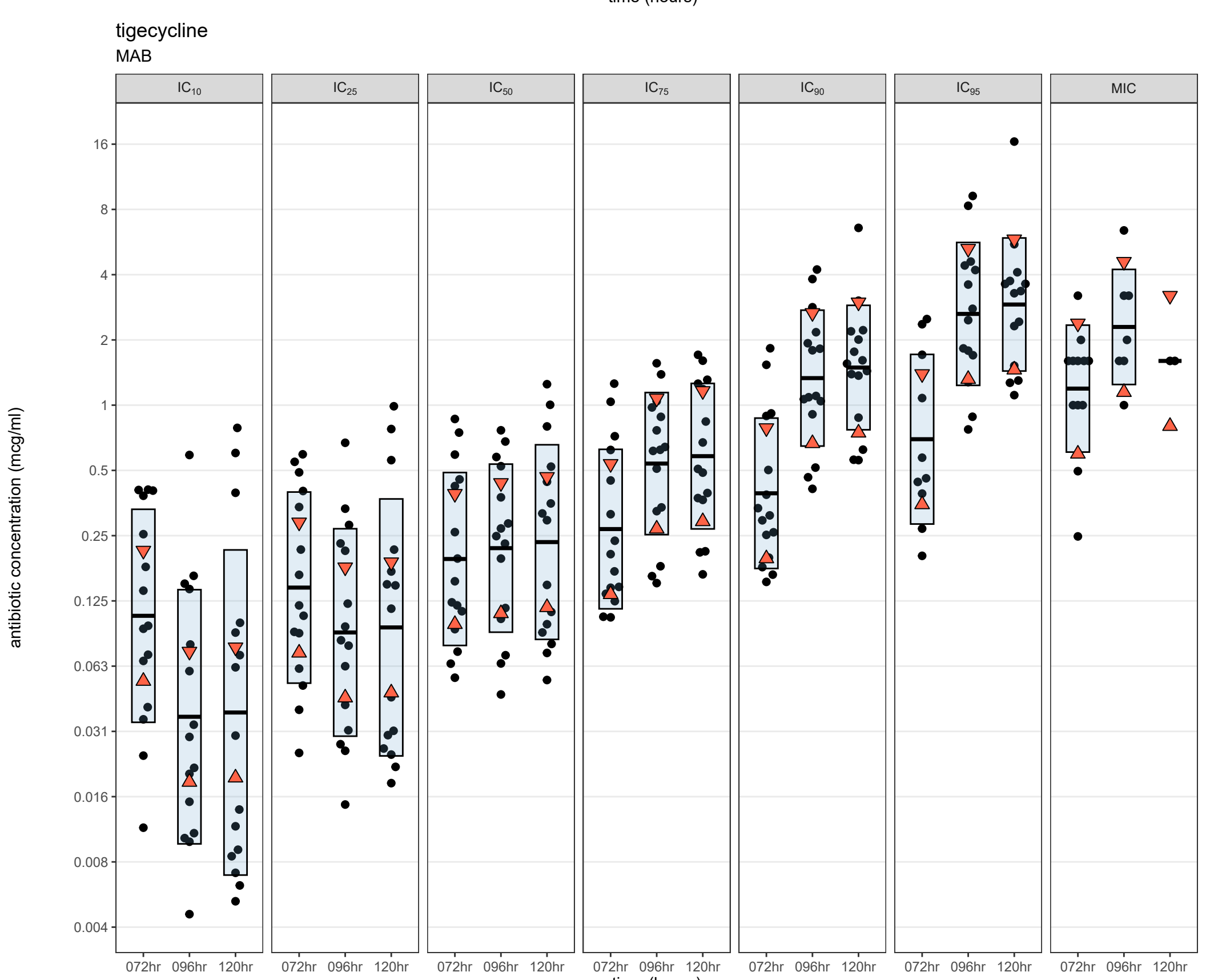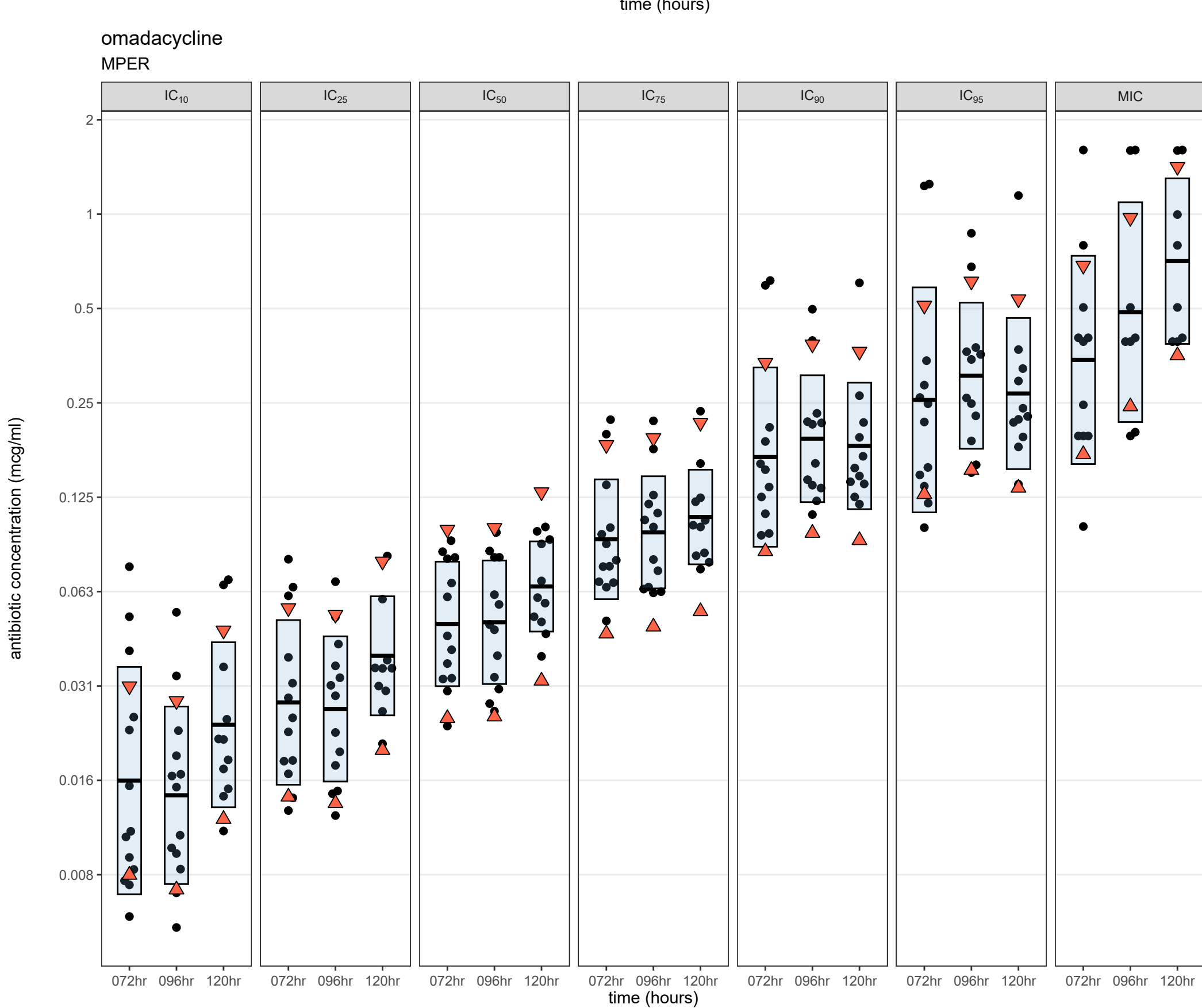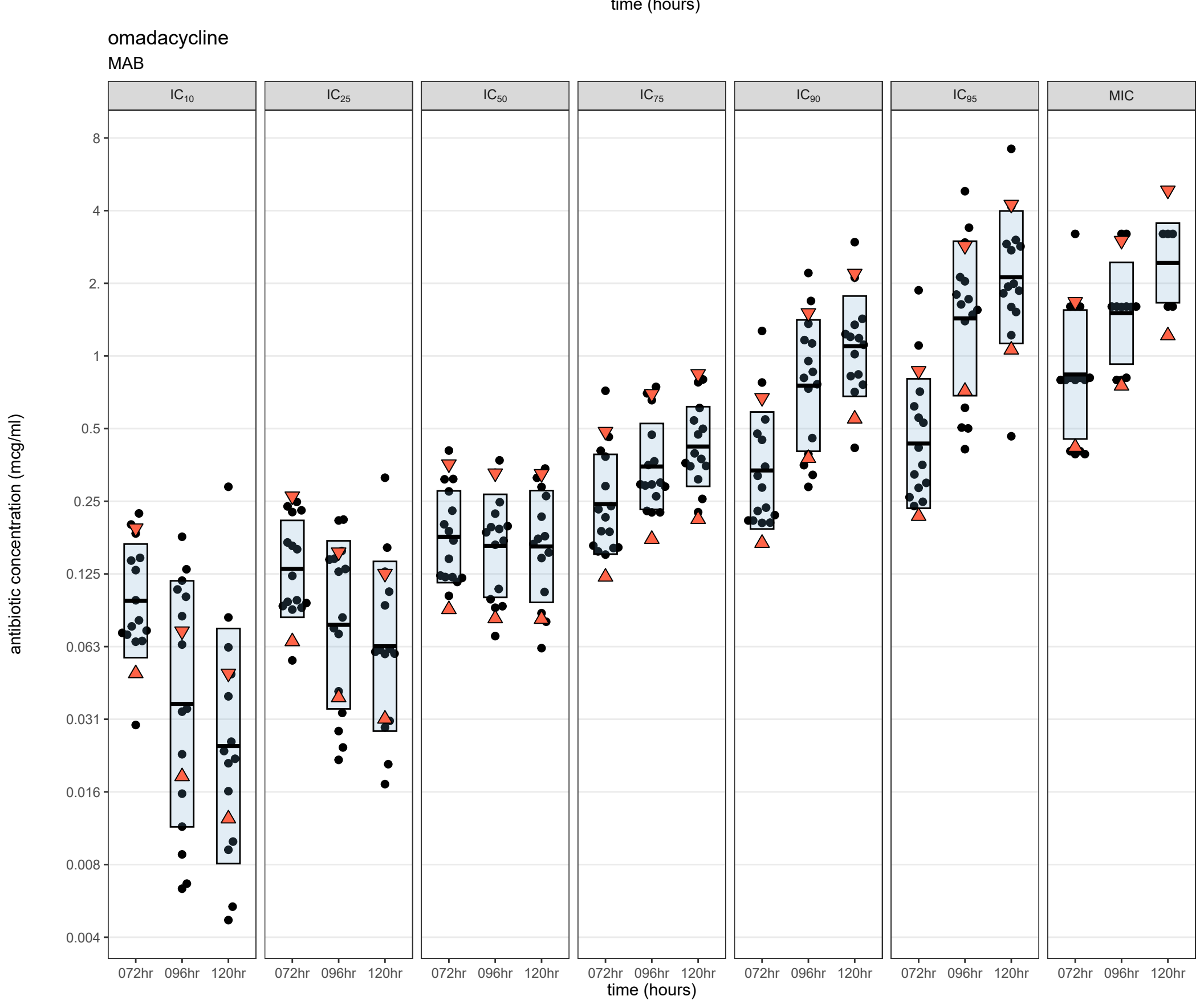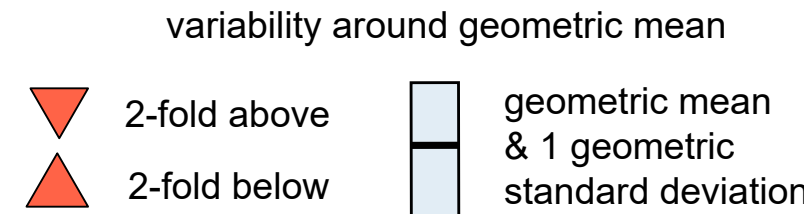

Supplement: Fig. S6 — IC and MIC values linezolid, tedizolid, tigecycline, and omadacycline. [file aac.01876-25-s0006.pdf]

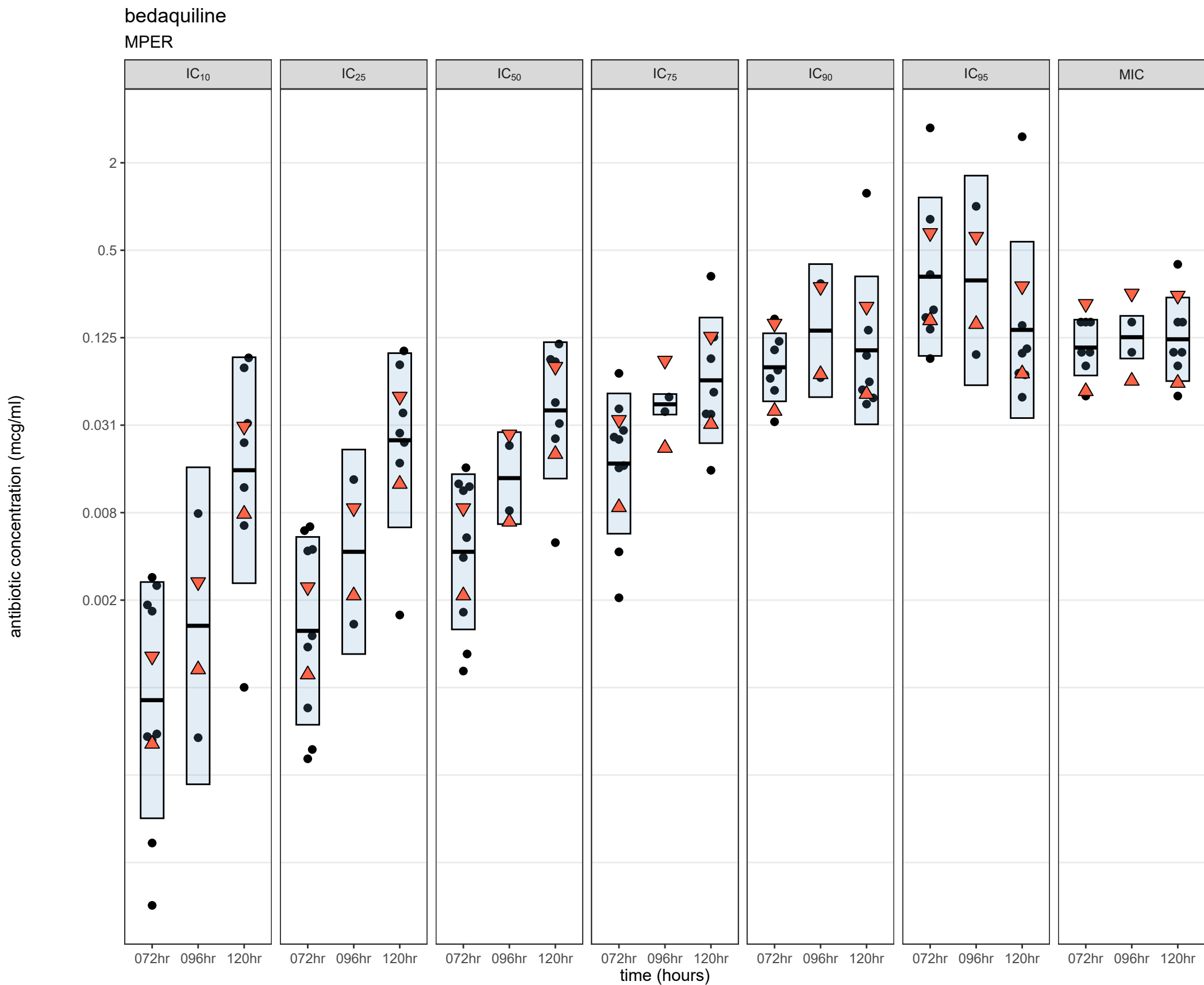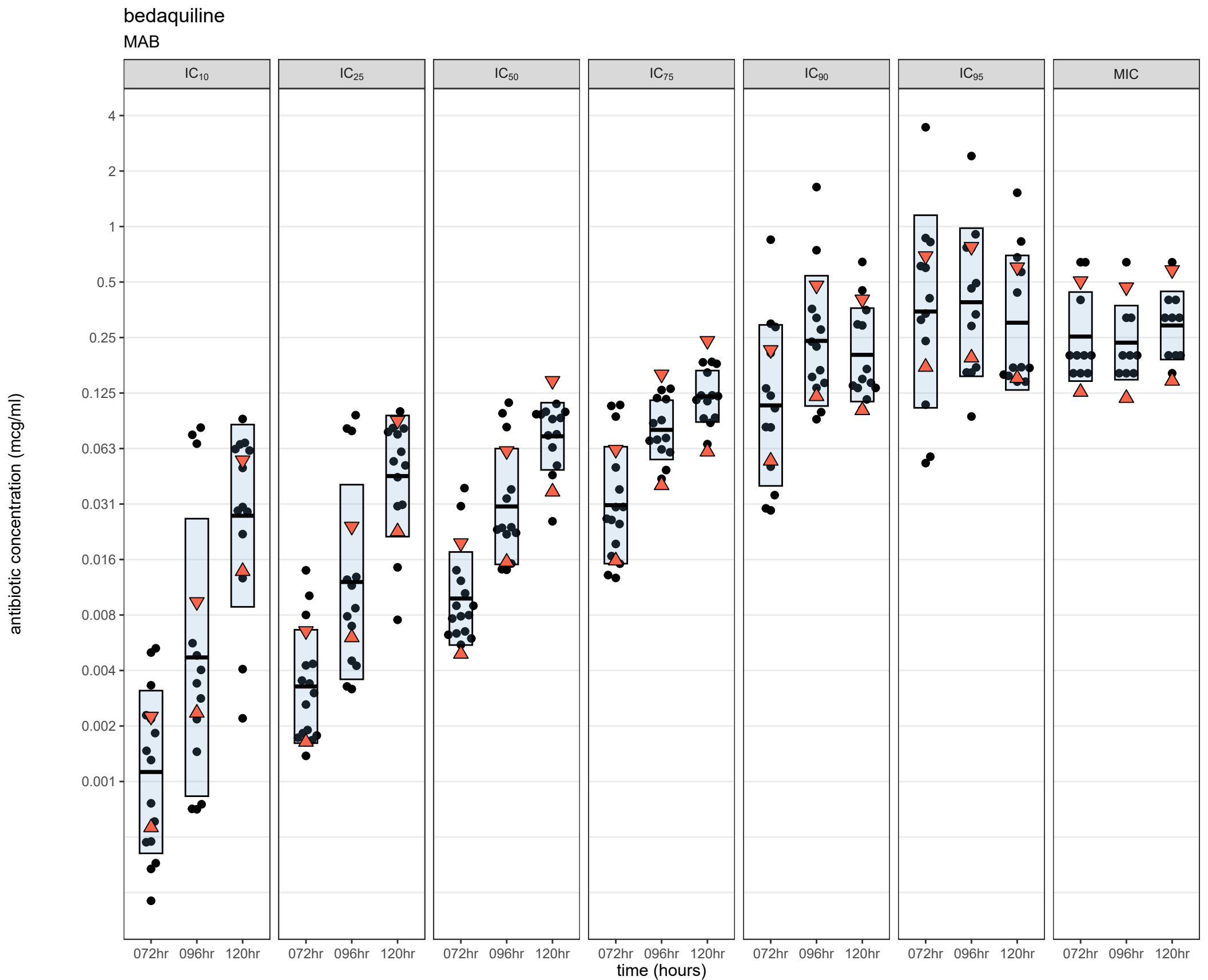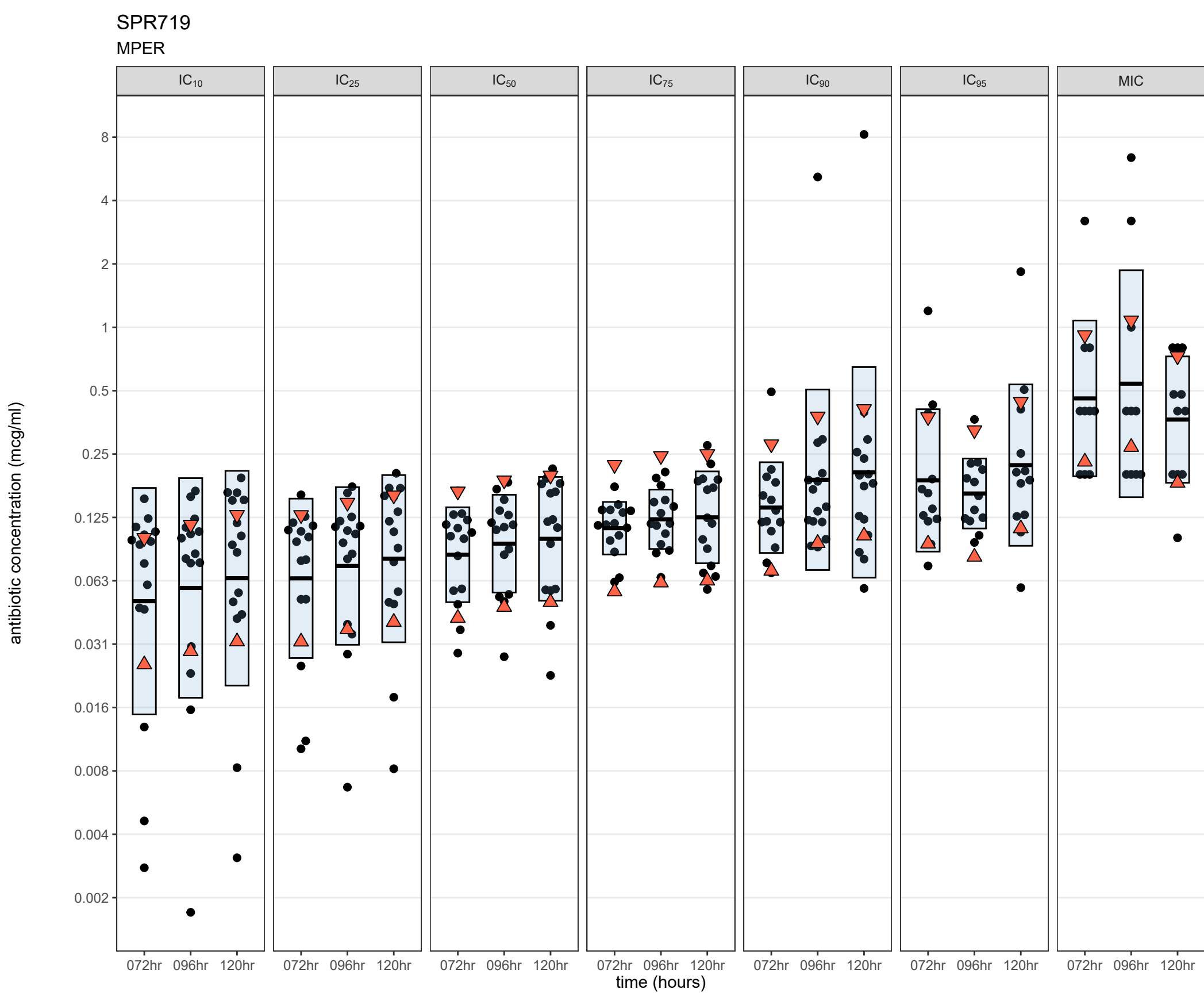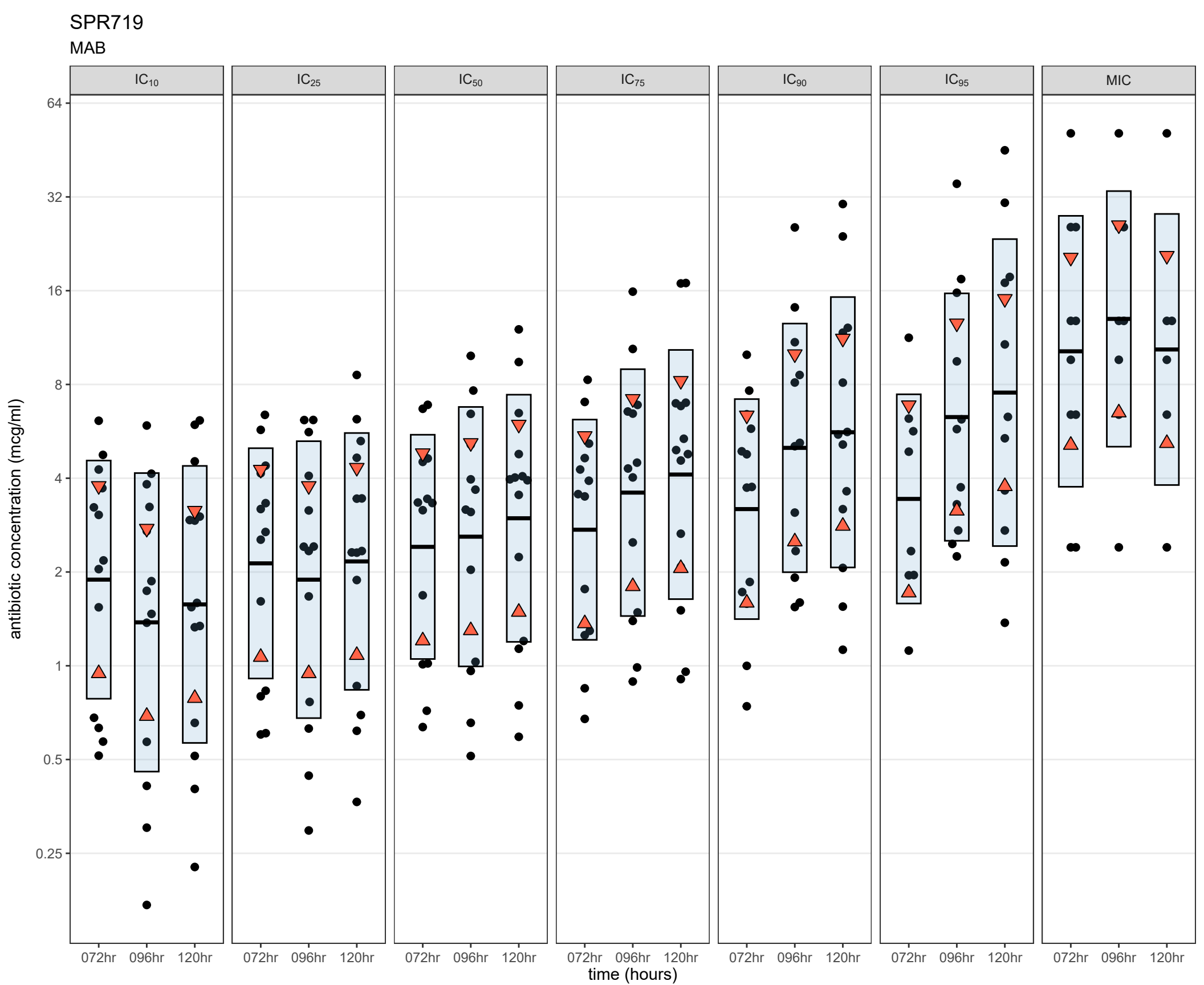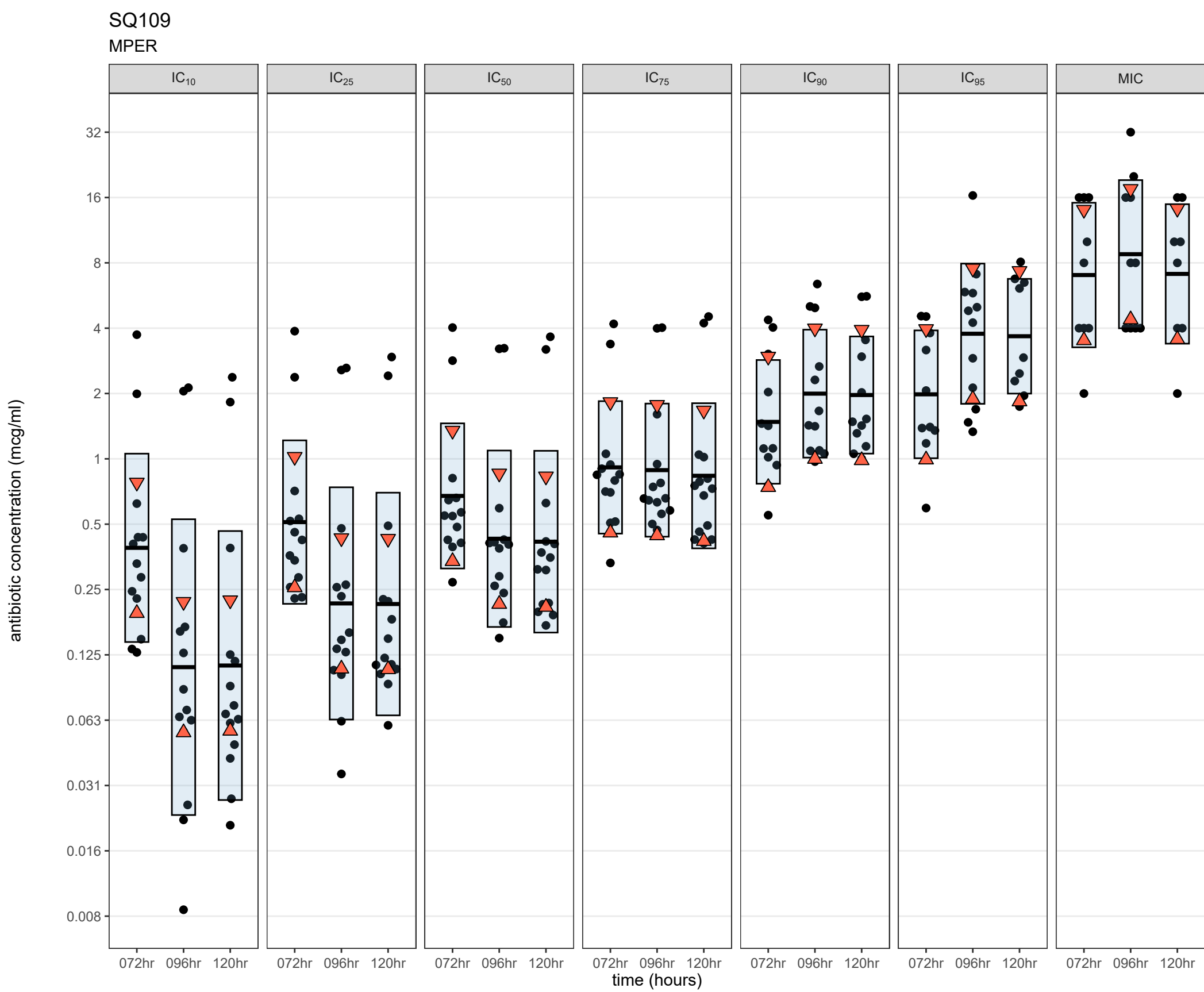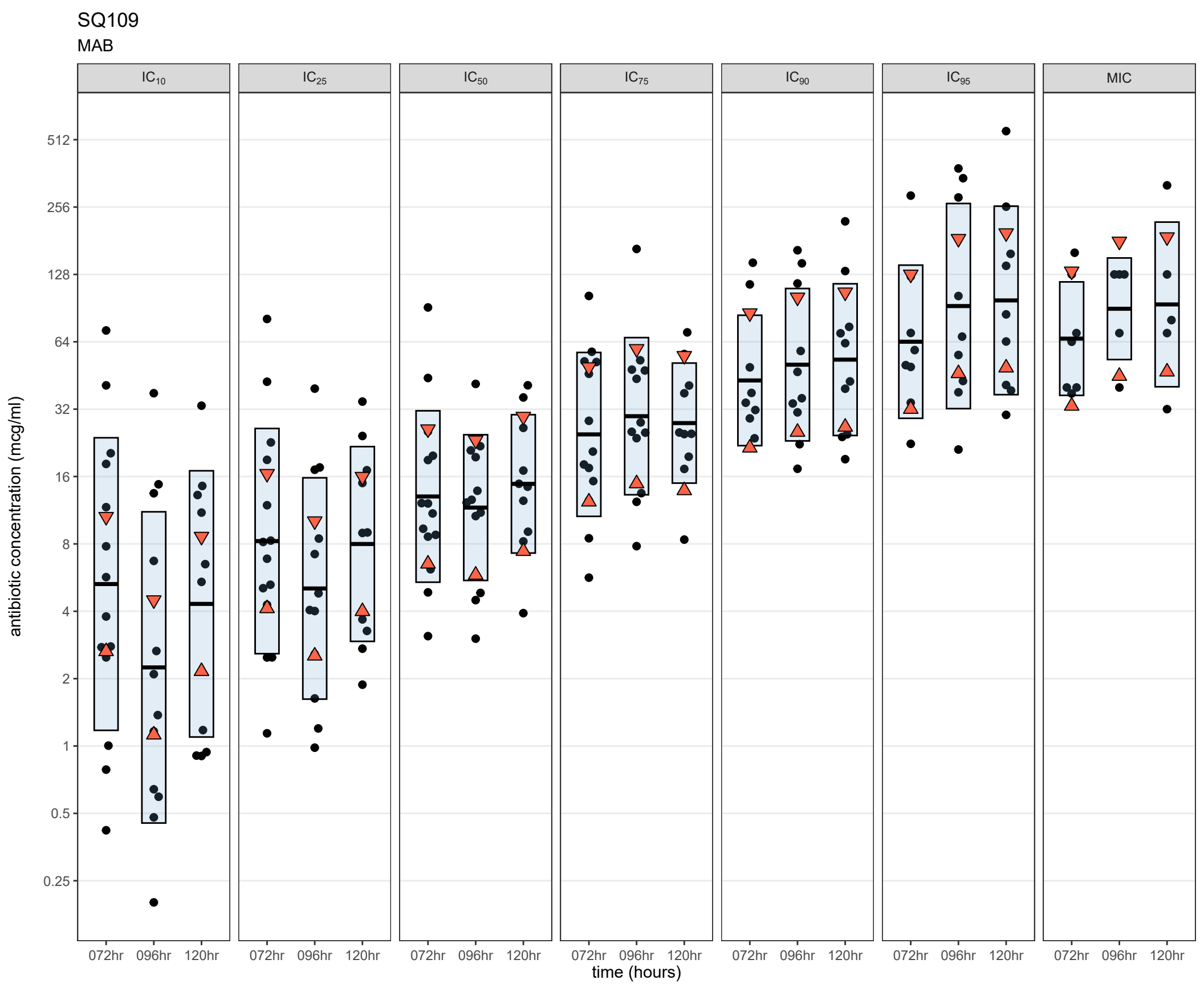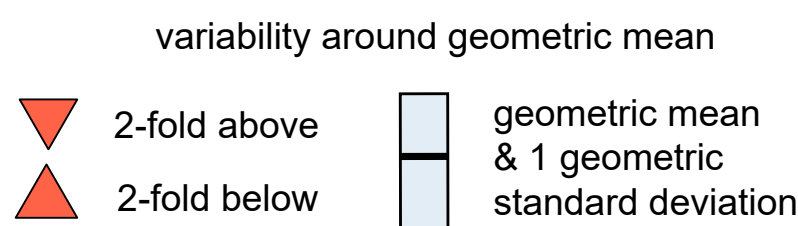

Supplement: Fig. S7 — IC and MIC values bedaquiline, SPR719, and SQ109. [file aac.01876-25-s0007.pdf]

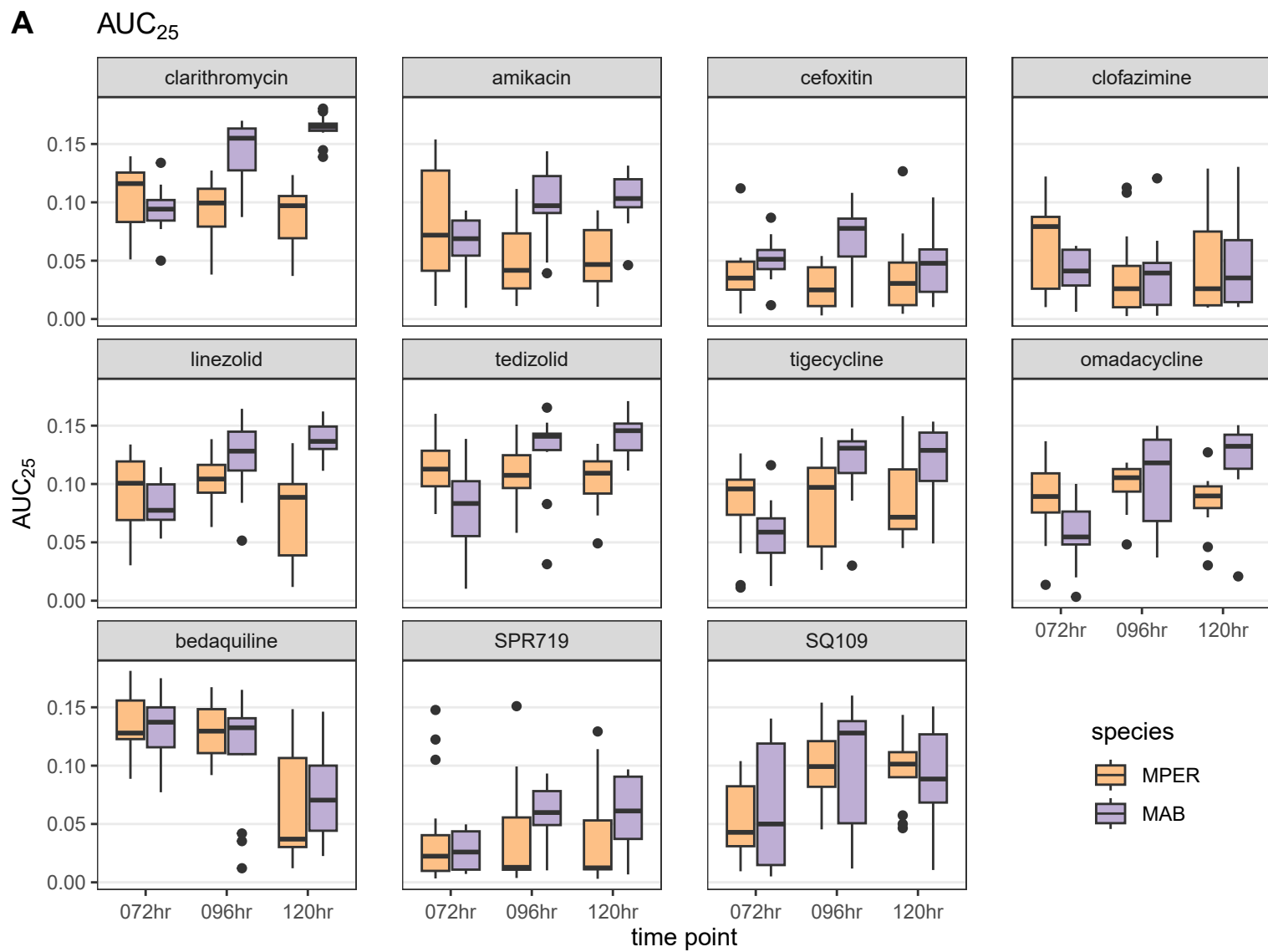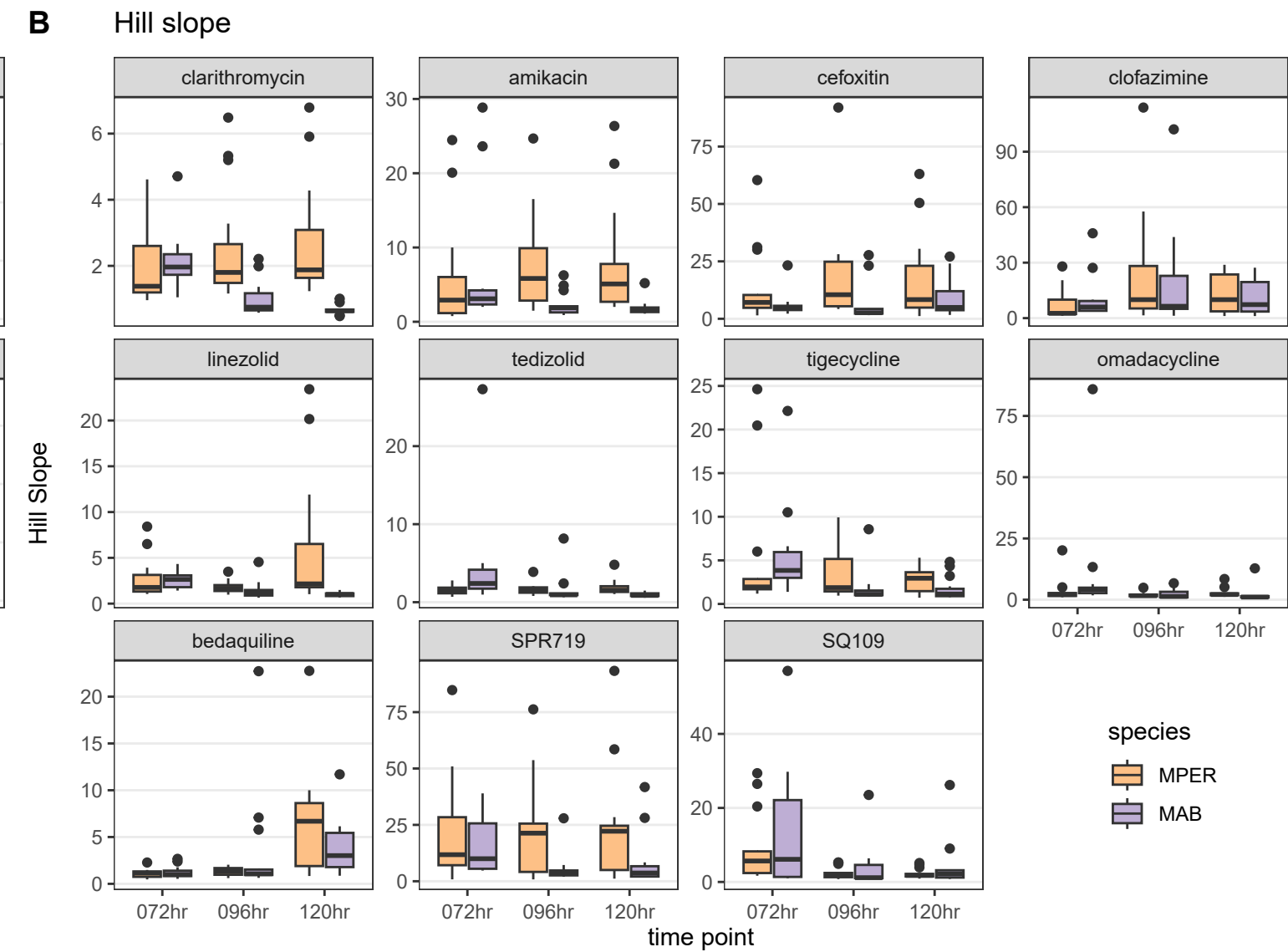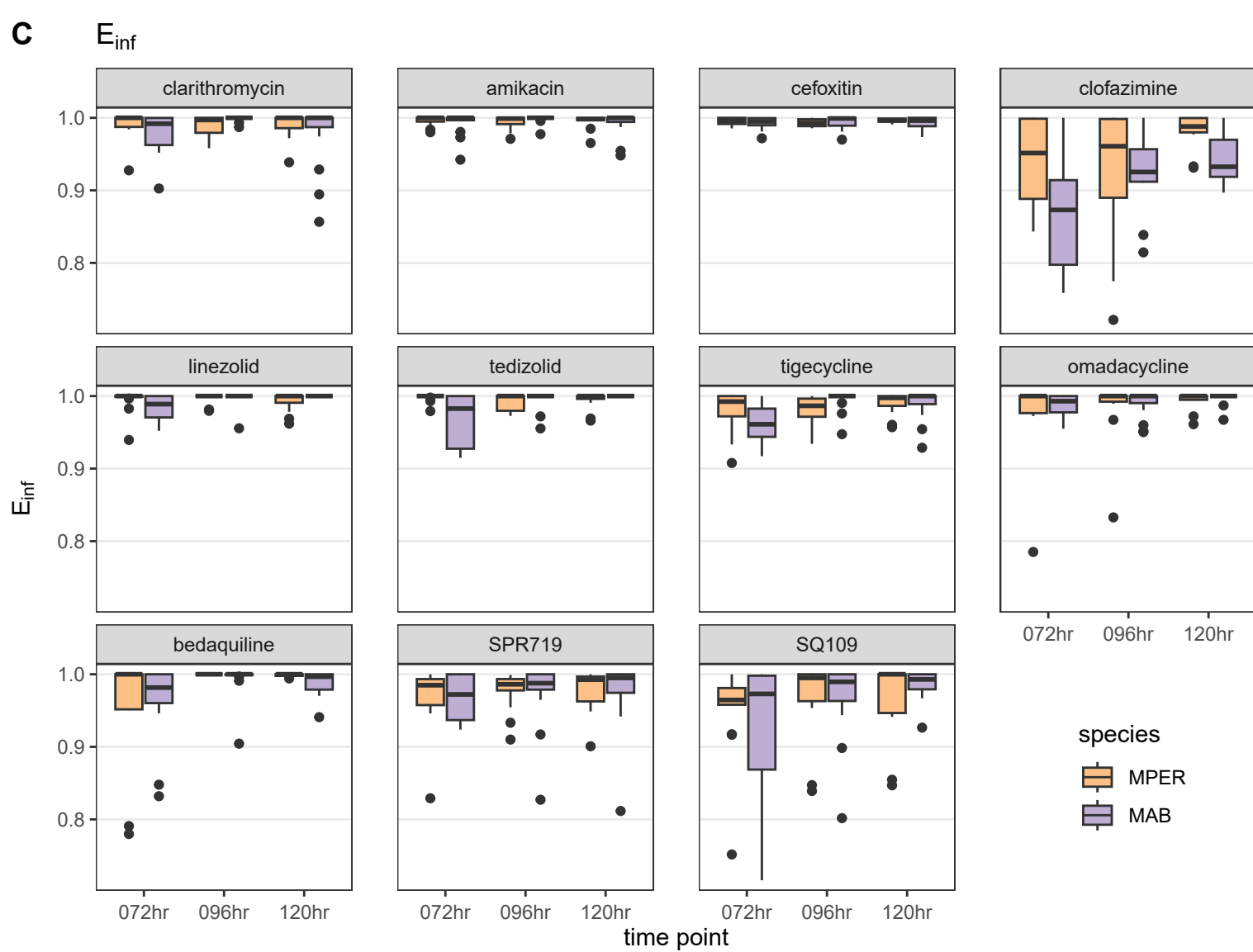

Supplement: Fig. S8 — Hill curve metrics. [file aac.01876-25-s0008.pdf]
