# Supplementary material for: Antibiotic dose-response curves can measure antibiotic activity against Mycobacterium abscessus and Mycobacterium peregrinum
Source: Antimicrob Agents Chemother. 2026 Apr 6;70(5):e01876-25. doi: 10.1128/aac.01876-25 (PMC13148060; doi:10.1128/aac.01876-25)
Supplement: Fig. S2 — Percentage of successful MIC measurements. [file aac.01876-25-s0002.pdf]

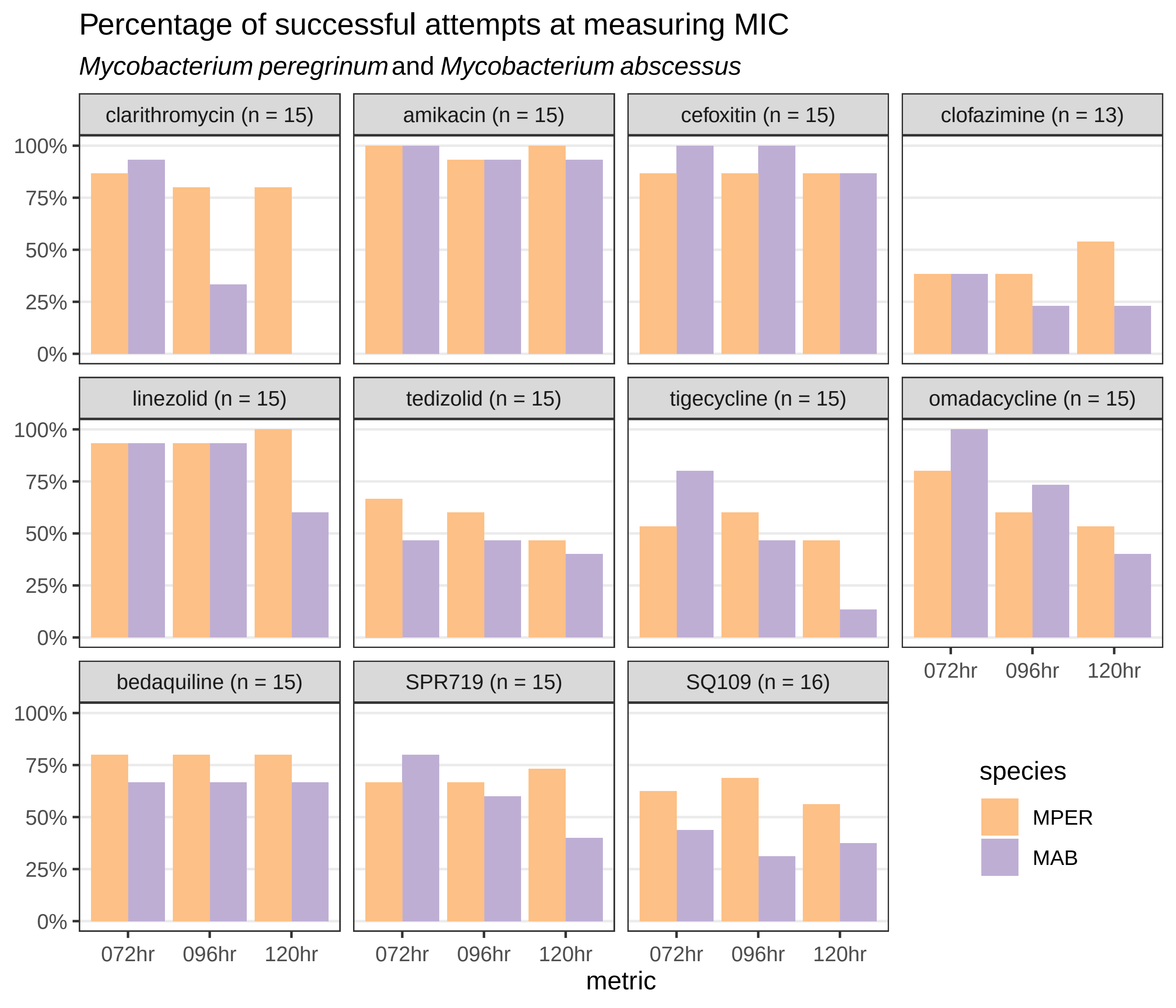
Percentage of successful attempts at measuring MIC
Mycobacterium peregrinum and *Mycobacterium abscessus*
clarithromycin (n = 15)
amikacin (n = 15)
cefoxitin (n = 15)
clofazimine (n = 13)
linezolid (n = 15)
tedizolid (n = 15)
tigecycline (n = 15)
omadacycline (n = 15)
bedaquiline (n = 15)
SPR719 (n = 15)
SQ109 (n = 16)
0%
25%
50%
75%
100%
072hr
096hr
120hr
metric
species
MPER
MAB
