# Supplementary material for: Antibiotic dose-response curves can measure antibiotic activity against Mycobacterium abscessus and Mycobacterium peregrinum
Source: Antimicrob Agents Chemother. 2026 Apr 6;70(5):e01876-25. doi: 10.1128/aac.01876-25 (PMC13148060; doi:10.1128/aac.01876-25)
Supplement: Supplemental material — Supplemental figure legends. [file aac.01876-25-s0009.docx]

## **Supplementary text**

## **MICs were hard to measure and higher than expected**

We defined the MIC for each antibiotic as the lowest concentration at which 100% growth inhibition was calculated. We were able to obtain the MIC for 66.3% of 990 attempts, with approximately 50% or lower success rates for one or more time points for clofazimine, tedizolid, tigecycline, omadacycline (for both MAB and MPER), and for SPR719 and SQ109 with MAB alone (Fig. S2). However, defining the MIC at 99% growth inhibition would lead to inclusion of 77% of isolates; at a threshold of 95% growth inhibition, 92% of replicates would be included. These support the idea that measuring extremes of growth inhibition is subject to variability not seen when measuring the IC_50_. Fewer MIC measurements were obtained for clarithromycin-MAB at 96 hours than at 72 hours, and none at 120 hours, likely due to inducible resistance.

For all antibiotic-bacteria pairs, while most replicate MIC measurements were within ±1 twofold concentration of the geometric mean, they did not meet CLSI criteria for essential agreement (Fig. S3). The spread of MIC measurements (measured using geometric mean and geometric standard deviation) was equivalent to the 2-fold range required by the CLSI for most antibiotics. For nearly all antibiotic-bacteria pairs, the geometric mean of the MIC exceeded the QC range (MPER) or CLSI breakpoints (MAB) (Fig. S3), suggesting that MICs obtained with this method cannot be compared with results from clinical laboratories.

We used the median coefficient of variation (the non-parametric equivalent of the coefficient of variation) to compare the variability across antibiotic-bacteria pairs (Fig. S4), with values less than 1.0 considered to be acceptable. The median coefficient of variation of all antibiotic-bacteria pairs was 1 or less, but this finding needs to consider the relatively low percentage of successful MIC measurements.

**IC values were measured across a wide dynamic range using a single stock concentration**

The drug dispenser can dispense volumes over a 100,000-fold range (0.013nL to 1000nL) using a single antibiotic stock concentration. This allowed the use of 13 doses to measure antibiotic activity over an 8000-fold range in concentration for both MPER and MAB in contrast to the 8 to 4000-fold range in concentration used in RAPMYCO2 Sensititre™ plate. We demonstrate this in Fig. S6, where the y-axis values for linezolid range from 0.031 mcg/ml for MPER to 512 mcg/ml for MAB. This is also seen with clarithromycin (Fig. 5, 0.002 to 256 mcg/ml), amikacin (Fig. S5 0.008 to 256 mcg/ml), tedizolid (Fig. S6, 0.002 to 256 mcg/ml), SQ109 (Fig. S7, 0.008 to 512 mcg/ml).

**Dose-response metrics AUC_25_ and Hill slope can be used to compare antibiotic activity**

As IC and MIC values cannot be used to compare and rank drugs, we calculated and compared the AUC_25_, Hill slope, and E_inf_ across all antibiotic-bacteria pairs (Fig. S8). Like the IC and MIC values, the median and interquartile ranges of these metrics were dependent on the *R^2^* values of the underlying dose-response curves. The AUC_25_ values and Hill slope values were inversely correlated, such that MAB has a higher AUC_25_ and lower Hill slope than MPER. The AUC_25_ values ranged between 0 and 0.20 and were higher for MAB than for MPER at 96 hours and at 120 hours. The AUC_25_ was similar between antibiotics belonging to oxazolidinone and tetracycline classes. The Hill slope, on the other hand, was lower for MAB than for MPER, with values ranging (except for outliers) between 0 and 30. There was more variability with the Hill slope values, and hence, we did not think it appropriate to make inferences about differences in Hill slope values between antibiotics. Our data suggest a higher AUC_25_ and lower Hill slope for a more antibiotic-resistant organism than for a less antibiotic-resistant organism. Except for clofazimine, all drugs had median E_inf_ values between 0.95 and 1, suggesting that complete inhibition of growth was possible.

## **Supplementary figure legends**

**Figure S1: Dose-response curve and derived metrics**

OD_600_ measurements are used to calculate growth inhibition (black circles) at each antibiotic concentration. The MIC is the antibiotic concentration at which 100% growth inhibition is first calculated. A dose-response curve is fit using a three-parameter Hill equation, with multiple metrics (in burgundy) derived from the curve. The IC_50_ and IC_90_ are the antibiotic concentrations that result in 50% and 90% growth inhibition, respectively. The AUC_25_ , Hill slope, and E_inf_ are metrics of potency. MIC, minimum inhibitory concentration; IC, inhibitory concentration; AUC_25_, area under the curve at 25% growth inhibition; maximum effect at infinite concentration, E_inf_ .

**Figure S2: Antibiotic-specific success in measuring MICs**

The percentage of successful minimum inhibitory concentration (MIC) measurements for each antibiotic (number of attempts in parentheses) is shown for *Mycobacterium peregrinum* (MPER, orange) and *Mycobacterium abscessus* (MAB, purple). The MIC was defined as the lowest antibiotic concentration at which 100% growth inhibition was calculated.

**Figure S3: Comparison of MIC measurements with published literature**

The minimum inhibitory concentrations (MICs) for each antibiotic at all time points (black points) for *Mycobacterium peregrinum* (A) and *Mycobacterium abscessus* (B), with the geometric mean represented by the horizontal bar. The MIC was defined as the lowest antibiotic concentration at which 100% growth inhibition was calculated. The upper and lower limits of ±1 2-fold range around the geometric mean are represented by the downward- and upward-pointing red triangles, respectively. The blue shaded area is 1 geometric standard deviation around the geometric mean. Dashed lines represent the antibiotic concentrations recommended for quality control ranges (MPER; orange and light blue) or susceptibility breakpoints (MAB; green, yellow, red) according to Clinical Laboratory and Standards Institution (CLSI) standards; for drugs without CLSI guidelines, published ranges were used (purple and lime green). There was only one antibiotic concentration for tedizolid and none for SQ109 or clofazimine. MPER, *Mycobacterium peregrinum*; MAB, *Mycobacterium abscessus* (B); hr, hours; MIC, minimum inhibitory concentration; S, susceptible; I, intermediate; R, resistant; min, minimum; max, maximum.

**Figure S4: The MICs showed acceptable variability**

The median coefficient of variation allows comparison of minimum inhibitory concentration (MIC) variability across antibiotics and time points and was calculated for *Mycobacterium peregrinum* (orange) and *Mycobacterium abscessus* (purple). We used a value of 1 or lower as an acceptable amount of variability, as it represents the value at which the median standard deviation is equal to the median and would be equivalent to ±1 2-fold range around the median. MPER, *Mycobacterium peregrinum*; MAB, *Mycobacterium abscessus* (B); hr, hours.

**Figures S5-S7: Variability of IC and MIC of antibiotics other than cefoxitin and clarithromycin**

The inhibitory concentration (IC) values for each replicate and the minimum inhibitory concentrations (MICs) for each at each time point (black points) for *Mycobacterium peregrinum* (MPER) and *Mycobacterium abscessus* (MAB), with the geometric mean represented by the horizontal bar. The upper and lower limits of ±1 2-fold range around the geometric mean are represented by the downward- and upward-pointing red triangles, respectively. The blue shaded area is 1 geometric standard deviation around the geometric mean. MPER, *Mycobacterium peregrinum*; MAB, *Mycobacterium abscessus* (B); hr, hours; IC_10,_ inhibitory concentration at 10% growth inhibition; IC_25_, inhibitory concentration at 25% growth inhibition; IC_50_, inhibitory concentration at 50% growth inhibition; IC_75_, inhibitory concentration at 75% growth inhibition; IC_90,_ inhibitory concentration at 90% growth inhibition; IC_95_, inhibitory concentration at 95% growth inhibition; MIC, minimum inhibitory concentration.

**Figure S8: Dose-response curve potency metrics allow comparison across antibiotics**

Boxplots of potency metrics: area under the curve at 25% growth inhibition (AUC_25_, A), the Hill slope (B), and E_inf_ (maximum effect at infinite concentration, C) are shown for each antibiotic for *Mycobacterium peregrinum* (MPER, orange) and *Mycobacterium abscessus* (MAB, purple). hr, hours.
