# Supplementary material for: Antibiotic dose-response curves can measure antibiotic activity against Mycobacterium abscessus and Mycobacterium peregrinum
Source: Antimicrob Agents Chemother. 2026 Apr 6;70(5):e01876-25. doi: 10.1128/aac.01876-25 (PMC13148060; doi:10.1128/aac.01876-25)
Supplement: Fig. S5 — IC and MIC values amikacin clofazimine. [file aac.01876-25-s0005.pdf]

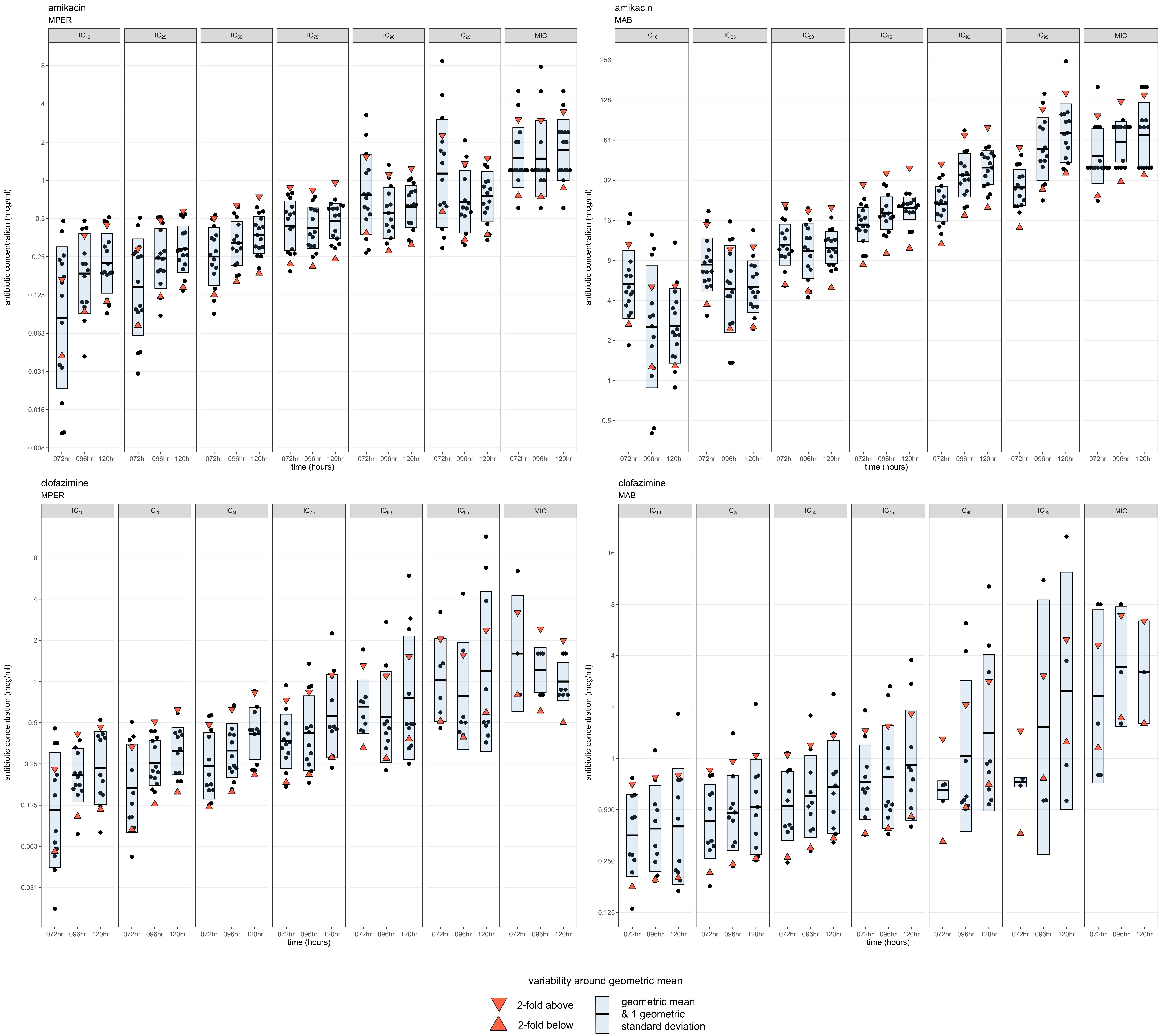
amikacin
MPER
amikacin
MAB
clofazimine
MPER
clofazimine
MAB
IC10
IC25
IC50
IC75
IC90
IC95
MIC
antibiotic concentration (mcg/ml)
072hr
096hr
120hr
time (hours)
variability around geometric mean
2-fold above
2-fold below
geometric mean
& 1 geometric
standard deviation
